# The functional diversity–productivity relationship of woody plants is climatically sensitive

**DOI:** 10.1002/ece3.11364

**Published:** 2024-05-02

**Authors:** Haoru Yan, Bernhard Schmid, Wubing Xu, Franca J. Bongers, Guoke Chen, Ting Tang, Zhiheng Wang, Jens‐Christian Svenning, Keping Ma, Xiaojuan Liu

**Affiliations:** ^1^ State Key Laboratory of Vegetation and Environmental Change Institute of Botany Beijing China; ^2^ University of Chinese Academy of Sciences Beijing China; ^3^ Department of Geography, Remote Sensing Laboratories University of Zurich Zurich Switzerland; ^4^ German Centre for Integrative Biodiversity Research (iDiv) Halle‐Jena‐Leipzig Leipzig Germany; ^5^ Centre for Crop Systems Analysis Wageningen University Wageningen The Netherlands; ^6^ Institute of Ecology and key Laboratory for Earth Surface Processes of the Ministry of Education College of Urban and Environmental Sciences, Peking University Beijing China; ^7^ Section for Ecoinformatics and Biodiversity, Department of Biology Aarhus University Aarhus Denmark; ^8^ Center for Biodiversity Dynamics in a Changing World (BIOCHANGE), Department of Biology Aarhus University Aarhus Denmark; ^9^ Zhejiang Qianjiangyuan Forest Biodiversity National Observation and Research Station Institute of Botany Beijing China

**Keywords:** biodiversity, climate change, forest restoration, functional traits, NPP, woody plant vegetation

## Abstract

Plot‐scale experiments indicate that functional diversity (FD) plays a pivotal role in sustaining ecosystem functions such as net primary productivity (NPP). However, the relationships between functional diversity and NPP across larger scale under varying climatic conditions are sparsely studied, despite its significance for understanding forest–atmosphere interactions and informing policy development. Hence, we examine the relationships of community‐weighted mean (CWM) and functional dispersion (FDis) of woody plant traits on NPP across China and if such relationships are modulated by climatic conditions at the national scale. Using comprehensive datasets of distribution, functional traits, and productivity for 9120 Chinese woody plant species, we evaluated the distribution pattern of community‐weighted mean and functional dispersion (including three orthogonal trait indicators: plant size, leaf morphology, and flower duration) and its relationships with NPP. Finally, we tested the effects of climatic conditions on community‐weighted mean/functional dispersion–NPP relationships. We first found overall functional diversity–NPP relationships, but also that the magnitude of these relationships was sensitive to climate, with plant size community‐weighted mean promoting NPP in warm regions and plant size functional dispersion promoting NPP in wet regions. Second, warm and wet conditions indirectly increased NPP by its positive effects on community‐weighted mean or functional dispersion, particularly through mean plant size and leaf morphology. Our study provides comprehensive evidence for the relationships between functional diversity and NPP under varying climates at a large scale. Importantly, our results indicate a broadening significance of multidimensional plant functional traits for woody vegetation NPP in response to rising temperatures and wetter climates. Restoration, reforestation actions and natural capital accounting need to carefully consider not only community‐weighted mean and functional dispersion but also their interactions with climate, to predict how functional diversity may promote ecosystem functioning under future climatic conditions.

## Graphical abstract

The relationship between plant functional diversity and productivity is climatic sensitive. Our results emphasize the importance of considering the interaction between climate and functional diversity in restoration and reforestation actions.

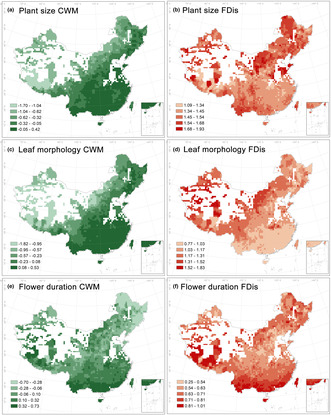

## 1 INTRODUCTION

Biodiversity–ecosystem functioning (BEF) relationship is essential for sustaining ecosystem functions to support human livelihood (Bongers et al., 2021; Durán et al., 2019; Huang et al., 2018; Isbell et al., 2015). Large‐scale BEF research is crucial for understanding diverse ecosystems, revealing regional‐scale relationships between biodiversity and productivity, and optimizing the predictions of future climate change impacts. Moreover, going beyond species diversity, functional diversity captures the subtler response of ecosystem functioning and service to global change (Diaz et al., 2007), operating through species combinations and species‐specific functional traits. The macroecological complementarity hypothesis (Burley et al., 2016; Chisholm et al., 2013) suggests that higher functional diversity would have greater magnitudes on ecosystem functions at a large scale, for example, the community‐weighted mean of leaf area (Li et al., 2020) and tree height (Wang et al., 2019) have been shown to promote primary productivity at 50 × 50 km and 100 × 100 km scale, respectively. However, enhancing woody vegetation productivity requires an exploration of how functional diversity (FD) and climate interactively influence productivity under varying climate conditions through community complementarity (Barry et al., 2019; Tilman et al., 1997) and selection effects (Grime, 1998). Studying the sensitivity of the relationship between functional diversity and productivity to climate change is crucial for broadly improving ecosystem productivity in restoration and reforestation and for quantifying the carbon acquisition of specific regions within natural capital schemes.

Community‐weighted mean (CWM) and functional dispersion (FDis) are two key multidimensional factors of functional diversity that represent community structure through complementarity effects and selection effects, respectively (Diaz et al., 2007). Community‐weighted mean measures the abundance‐weighted mean trait value of all coexisting species in the community, while functional dispersion reflects the variation of single or multivariate functional trait values among species. A positive community‐weighted mean and productivity relationship indicates that dominant species traits can promote productivity through biodiversity selection effects (Li et al., 2020; Wang et al., 2019), as proposed by mass‐ratio theory (Grime, 1998). A positive functional dispersion and productivity relationship suggests that the coexistence of species with different functions in a community can promote ecosystem functions, such as productivity, through resource partitioning, abiotic facilitation, and biotic feedback, as proposed in niche complementarity theory (Barry et al., 2019; Tilman et al., 1997). Community‐level research consistently indicated a general positive functional diversity–productivity relationship (Bongers et al., 2021; Durán et al., 2019) and the large‐scale studies showed that the community‐weighted mean of tree height (Wang et al., 2019) and leaf area (Li et al., 2020) can predict ecosystem primary productivity, as idea derived from macroecological complementarity hypothesis (Burley et al., 2016; Chisholm et al., 2013). As research transitions from local‐scale studies to large‐scale analyses, the relationship between functional diversity and productivity may influenced by scale effects and various environmental factors. Thus, there is still a need to understand whether the multiple dimension function of community‐weighted mean and functional dispersion additionally drive productivity at a large scale.

The functional diversity–productivity relationship can be influenced by both abiotic and biotic factors. For abiotic factors, more positive effects of functional diversity on productivity have been observed in colder or drier regions than in warmer (Paquette & Messier, 2011) or wetter (Ratcliffe et al., 2016) regions, because positive diversity effect can better promote productivity through architectural or structural modifications in stressful (cold or drought) conditions, as proposed by the stress gradient hypothesis (Maestre et al., 2009; Wright et al., 2017). In warmer regions, such as tropical forests, the saturating effect of diversity may lead to functional redundancy, potentially diminishing the positive functional diversity–productivity relationship (van der Sande et al., 2017). Therefore, climatic complexity leads to diverse local conditions, further increasing the uncertainty of the positive relationship between functional diversity and productivity at a large scale. For biotic factors, stand biomass and plant maximum size could significantly drive ecosystem productivity and the effects increase with temperature, precipitation, growing season length, and plant age at a large spatial scale, an idea derived from metabolic scaling theory (Michaletz et al., 2014, 2018). Assessing the influence of functional diversity on productivity becomes challenging when biomass has a more immediate impact on productivity. Furthermore, considering the environmental limitations of current local‐scale studies, extrapolating how the relationship between functional diversity and productivity varies on a larger scale remains intricate.

Climate can directly and indirectly (through modulating functional diversity) affect productivity (Conradi et al., 2020; Li et al., 2020), making it feasible to optimize productivity through enhanced functional diversity under varying climatic conditions. Both current and past climates determine species pool composition and richness through environmental filtering (Conradi et al., 2020), which directly affects functional diversity. Paleoclimatic instability suggests that significant climate changes over decades to millions of years have influenced functional diversity through evolutionary lags and filtering processes, such as causing non‐random extinctions of frost‐tolerance traits (Svenning, 2003) and shaping the distribution pattern of dispersal traits (Kissling et al., 2012). Temperature and temporal heterogeneity of soil‐water availability (Gherardi & Sala, 2015) also indirectly influence functional diversity by filtering functional traits, such as leaf area (Diaz et al., 2007). Climate conditions directly impact productivity, with paleoclimatic instability shaping local species composition and influencing overall productivity (Conradi et al., 2020). Temperature and precipitation could affect productivity by controlling plant metabolic rate (Boisvenue & Running, 2006) and growth rate, especially under extreme droughts (Anderegg et al., 2015). Additionally, water‐use efficiency may increase with elevated CO_2_ in future climates (Berg & McColl, 2021), reducing soil water limitations on plant productivity and thereby positively impacting overall productivity. Therefore, climatic conditions may affect functional diversity and productivity independently, but how climatic conditions modulate the functional diversity–productivity relationships are still unclear.

To explore the relationship between functional diversity and net primary productivity (NPP) of woody vegetation and how this relationship is modified by climate at a large spatial scale, we use large species distribution and functional trait datasets encompassing 9120 woody plants across China. Eight functional traits were categorized into three orthogonal trait indicators, representing plant size, leaf morphology, and flower duration. Tree height, fruit size (fruit length and width), and seed mass were indicative of the size of whole or part plants (Diaz et al., 2016) within a community. Leaf morphology traits, such as leaf area and petiole length, play a crucial role in enhancing productivity by optimizing radiant energy capture (Reich et al., 1997; Wright et al., 2004), gas exchange, and light distribution (Perez et al., 2018). Flower traits, such as flower duration, indirectly affect productivity through reproductive strategy and pollination success (Marchin et al., 2015). By calculating community‐weighted mean and functional dispersion for these three orthogonal trait indicators, we aimed to address two key questions within 50 × 50 km grid cells: (1) whether the functional diversity–NPP relationships exist and if it would be modulated by climatic conditions; (2) whether the climatic conditions would affect NPP through functional diversity. We predicted that: H1, positive general relationships between functional diversity (community‐weighted mean or functional dispersion) and NPP in Chinese woody plant communities but the magnitude of these functional diversity–NPP relationships could be modulated by climatic conditions for the following reason. The mass‐ratio hypothesis (Grime, 1998) suggests that the functional traits of abundant species, that is community‐weighted mean, may promote NPP by increasing the dominant species traits value. Additionally, the coexistence of species with different traits within communities, that is functional dispersion, may increase community resource extraction, consequently increasing NPP. Thus, consistent with the local scale, the positive functional diversity–NPP relationship persists at a large scale even under various climate conditions, as posited by the macroecological complementarity hypothesis (Burley et al., 2016; Chisholm et al., 2013). Furthermore, stressful climatic conditions may increase the positive interactions of resource partitioning traits, as proposed by the stress‐gradient hypothesis (Maestre et al., 2009). Conversely, in more favorable climatic conditions, resource competition could be stronger, potentially decreasing the positive functional diversity–NPP relationship. H2: climatic conditions could also affect NPP indirectly through functional diversity because climatic conditions can directly affect functional diversity, which in turn may affect NPP.

## 2 MATERIALS AND METHODS

### 2.1 Functional traits

The functional trait data for woody species were sourced from the Chinese plant functional traits database, which was compiled from “Flora of China” (http://www.iplant.cn/foc/) and other books and papers. The functional traits database contains 39,757 species, representing 3407 genera and 299 families. For the purposes of this study, only woody species (trees and shrubs) were considered, excluding woody lianas, climbers, and scandent shrubs to avoid overestimating the functional diversity of tree height. The herbaceous plants were excluded because when compared to herbaceous plants, woody plants contribute significantly to diverse habitats, and exhibit superior ecological attributes and a broader range of functions within ecosystems (Dixon et al., 1994; Pan et al., 2013). Woody plants, with deep root systems and soil carbon storage capacity, play a pivotal role in enhancing soil stability and contribute significantly to global carbon cycling and climate regulation. Moreover, the longevity of woody plants sustains ecological functions over extended periods, supporting biodiversity and providing effective protection against environmental change. Excluded exotic and cultivated species were further excluded using “Flora of China” (http://www.iplant.cn/foc/) and the “International Plant Name Index” (IPNI, https://www.ipni.org/). Taxonomic names of all woody species were standardized through the “Taxonomic Name Resolution Service” (https://tnrs.biendata.org/, TNRS V5.0), and taxa below the species level were merged into the corresponding species. Consequently, the dataset comprised 9120 woody species, belonging to 1018 genera and 162 families.

For each species, we extracted eight quantitative traits: tree height, leaf length, leaf width, petiole length, fruit length, fruit width, seed mass, and flower duration. To minimize the effects of different shapes, the leaf area was represented as 2/3 × leaf length × leaf width (Li et al., 2020). Fruit length represented fruit size, while fruit width was used as a substitute when length data were missing. To address missing trait data, we employed genus mean value filling (Table S1) and 1000‐runs predictive mean matching (PMM) of multivariate imputation using the “mice” package (Groothuis‐Oudshoorn, 2011). We conducted varimax rotation principal component analysis (PCA) on the species traits (log‐transformed) to obtain three orthogonal axes using the “psych” package in R (Revelle, 2021) for both the original and after‐filling functional traits data. The similarity in the functional traits PCA distribution before and after filling suggests the robustness of the functional trait filling and the dataset after filling remains representative of the original trait dataset (Figures S1 and S2; Table S2 and S3). The first three axis partitioned these traits into three orthogonal axes related to plant size (PC1), leaf morphology (PC2), and flower duration (PC3) (Figure S2; Table S3). As multiple‐module traits, tree height, fruit size, and seed mass can represent the size of a whole plant (Diaz et al., 2016), directly influencing whole‐plant resource acquisition in a community and indirectly affecting dispersion (Galetti et al., 2013) and the survival of subsequent generations (Wang et al., 2019; Westoby et al., 1996). Leaf area and petiole length can represent leaf morphology, driving productivity by promoting plant growth. Leaf area expands the surface for sunlight absorption and promotes productivity by enhancing the light capture ability (Li et al., 2020; Reich et al., 1997). Petiole length optimizes gas exchange and transpiration processes within individual leaves, minimizing mutual shading, and optimizing light distribution within the plant crown (Perez et al., 2018), potentially affecting community productivity. Flower duration describes the indirect impact of phenology on reproductive strategy, as a longer flower duration tends to increase pollination success rates, consequently boosting fruit production and the productivity of subsequent generations (Marchin et al., 2015).

### 2.2 Species distribution data

Species distribution data at the county level were sourced from the Chinese Vascular Plant Distribution Database (Xu et al., 2018), compiled from over 1000 published floras, checklists, inventory reports, and more than 6 million specimens available through the Chinese Virtual Herbarium (CVH, http://www.cvh.ac.cn) and the National Specimen Information Infrastructure of China (NSII, http://www.nsii.org.cn/2017/home.php). The distribution maps were already transformed into binary (0/1) species distribution data at the county level. In total, we collected data for 9120 woody species, resulting in a comprehensive dataset with 437,184 distribution records at the county level.

### 2.3 Functional diversity estimation

County‐level functional diversity for the three PCA axes was estimated using county‐level species presence data and species‐level functional traits data (Figure S3). Community‐weighted mean is defined as the mean value of tree functional traits across all woody species coexisting in a community. Additionally, community‐weighted mean was based on the scores of the first, second, and third PCA axes (Table S3), representing plant size, leaf morphology, and flower duration within each community. Functional dispersion is defined as the mean distance of trait values of all co‐occurring species to the mean trait value in a community (Laliberte & Legendre, 2010). Functional dispersion quantifies niche complementarity (Ratcliffe et al., 2016), representing the potential complementarity during growth within each community. Compared to other functional diversity indices, such as functional richness, functional evenness, and functional divergence, functional dispersion considers the individual trait's value difference loaded on each PC. All individual traits were z‐transformed (mean = 0 and SD = 1) before calculating functional dispersion.

Both community‐weighted mean and functional dispersion were computed using the FD package (Laliberte & Legendre, 2010). To minimize the influence of county size on functional diversity estimates, county‐level functional diversity (community‐weighted mean and functional dispersion of three functional groups) was transformed into equal‐area grid cells with a size of 50 × 50 km using the “exactextractr” package in R (Baston, 2020) with an Albers equal‐area projection. Community‐weighted mean or functional dispersion values in each grid cell were defined as their mean value weighted by the fraction covered by the counties in each grid cell. The terms “grid cell” and “community” are used interchangeably. While most relationships between functional diversity factors are weak, we observed that leaf morphology community‐weighted mean has a positive correlation with plant size community‐weighted mean and a negative association with leaf morphology functional dispersion (Figure S4).

### 2.4 Woody vegetation NPP and forest aboveground biomass

Net primary productivity (NPP) data for the years 2000–2015 at a resolution of 1 × 1 km were obtained from the Numerical Terradynamic Simulation Group (http://www.ntsg.umt.edu). NPP was estimated using the C5 MOD17 algorithm applied to MODIS data (Zhao et al., 2005). For each grid cell, NPP was calculated as the mean value over the entire study period. In addition to NPP, forest aboveground biomass (AGB) was extracted based on over 8000 ground inventory records collected after 2000 from published literature. This information was combined with Geoscience Laser Altimeter System (GLAS)/Ice, Cloud, and Land Elevation Satellite (ICESat) data, optical imagery, climate surfaces, and topographic data (Su et al., 2016). Woody vegetation NPP and forest aboveground biomass for forest and shrub land were further extracted from the GlobeLand30 dataset (http://www.globallandcover.com/), which includes land covered by more than 30% of trees or shrubs (Figure S5a). The mean of NPP values and the sum of AGB values were aggregated into 50 × 50 km grid cells.

### 2.5 Climatic conditions

Climatic variables for the present (1970–2000) were extracted from the World Clim 2.1 database at a resolution of 1 × 1 km. Paleotemperature velocity (PTV), ranging from 0.06 to 50.85 g C m^−2^ year^−1^, served as a measure of temperature‐change velocity along spatial gradients since the last glacial maximum (LGM). PTV is associated with the migration rate of species prompted by climate change (Loarie et al., 2009). The displacement rate of climatic conditions during the LGM along spatial gradients was obtained following the method of Sandel et al. (2011), based on data simulated by CCSM4 and MIROC‐ESM from World Clim (Figure S5b). Mean annual temperature (MAT, bio1, ranging from −23.19°C to 27.15°C) and mean annual precipitation (MAP, bio12, ranging from 9 to 4870 mm year^−1^) were considered, as they strongly influence NPP, particularly in drier and colder ecosystems (Figure S5c,d).

Multicollinearity among the three climatic variables was weak, as indicated by variance inflation factors (VIFs) < 5. Although the MAT‐MAP correlation was somewhat strong, the PTV exhibited a weak relationship with MAT or MAP (Figure S4). All climatic variables were aggregated into the same 50 × 50 km grid cells as presented above, using grid mean values.

### 2.6 Data analyses

First, the geographical patterns of community‐weighted mean and functional dispersion for the three trait groups were estimated. To eliminate the effects of low species richness on functional diversity–NPP relationships, counties with fewer than 10 species were excluded. Grid cells with less than 50% forest area (<1250 km^2^) or no NPP or functional diversity values were also excluded. In the end, 2732 grid cells were included in the analyses.

Second, the relationship between community‐weighted mean, functional dispersion values for each trait group (plant size, leaf morphology, and flower duration), and NPP were examined. The analysis also investigated whether climatic conditions could modulate functional diversity–NPP relationships (H1). Multiple linear regression models (LM) were conducted, testing the associations between community‐weighted mean and functional dispersion values per trait group and NPP under current and past climatic conditions. NPP has been square root transformed in the following analysis. Each model included one climatic variable, one functional diversity index, and their interaction. To account for spatial autocorrelations, spatial simultaneous autoregressive (SAR) error models were built using the “spatialreg” package in R (Kissling & Carl, 2008). Analysis of step‐neighbor distances from 100 km to 1000 km indicated that a neighbor distance of 100 km resulted in a minimal Akaike information criterion (AIC) value. Moran's I of residuals of the SAR error models suggested that spatial autocorrelation had been removed successfully (Figure S6).

Third, structural equation models (SEMs) were fitted using the “piecewiseSEM” package in R (Lefcheck, 2016) to assess the direct (paths from climatic variables to NPP) and indirect effects (paths via community‐weighted mean and functional dispersion of three trait groups) of climatic variables on NPP across China (H2). Standardized path coefficients and variation of functional diversity and NPP were also estimated and the model with the lowest Akaike information criterion (AIC) was chosen as the best model. If model differences in AIC were < 2, we considered the simpler model as the better one. The path analysis was conducted together and separately for each trait group. Furthermore, to demonstrate that the NPP could be affected by functional diversity, rather than just the effects of biomass, an SEM model using AGB as endogenous variable was built in the same way as NPP. All statistical analyses were conducted in R 3.6.1 (R Core Team, 2019).

## 3 RESULTS

### 3.1 Geographical patterns of functional diversity and NPP

**FIGURE 1:**
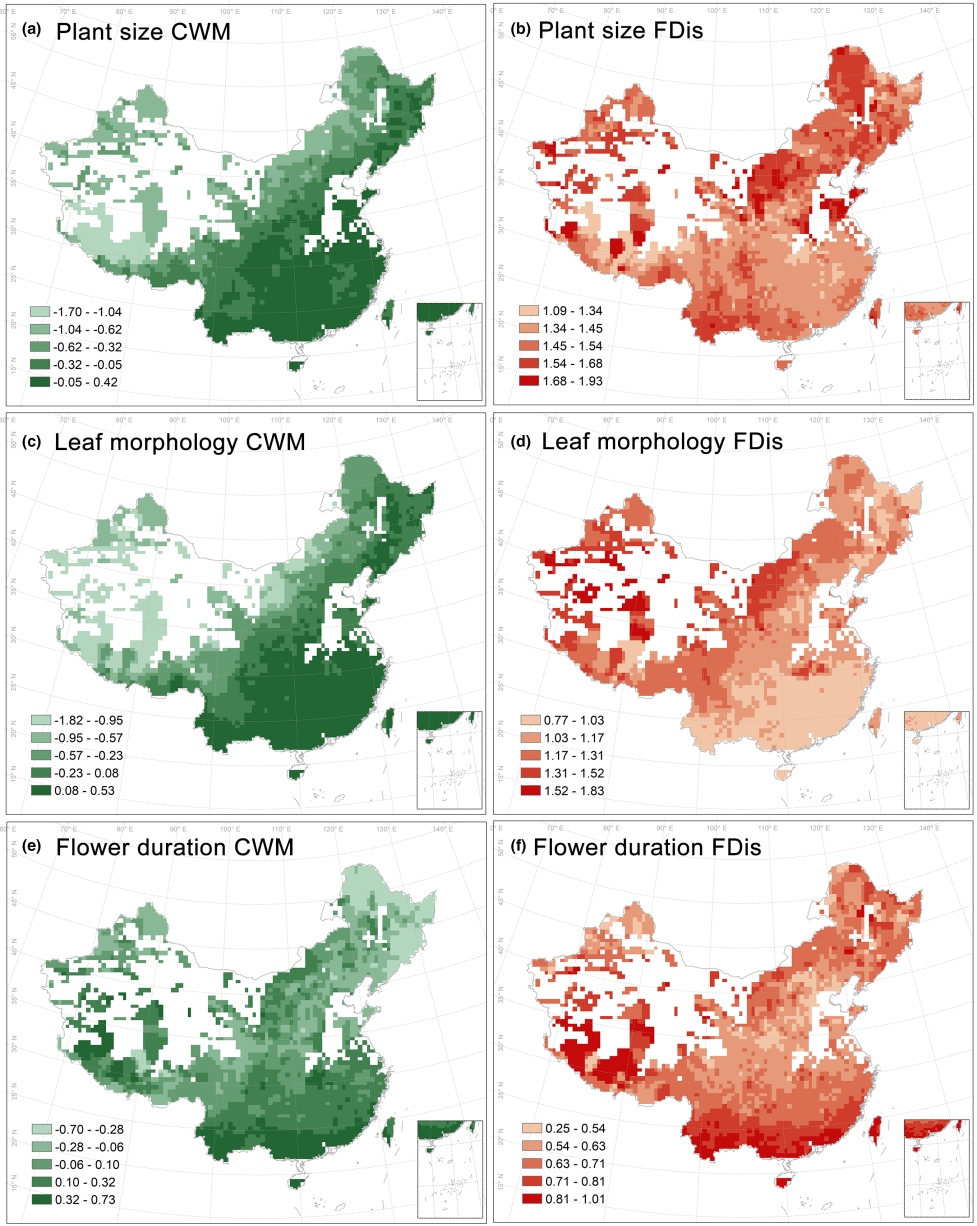
Spatial patterns of functional diversity based on 9120 woody species across China at the spatial scale of 50 × 50 km. The community‐weighted mean of plant size (a), leaf morphology (c) and flower duration (e). Functional dispersion of plant size (b), leaf morphology (d) and flower duration (f). The white grids indicate cells containing less than 10 woody species or without woody species productivity; these were excluded from our analysis. CWM, community‐weighted mean; FDis, functional dispersion.

In warm and wet regions, woody plant species exhibit large plant size and leaf morphology community‐weighted mean (Figure 1a,c). Conversely, in cold regions, there are large plant size and leaf morphology functional dispersion values (Figure 1b,d). The community‐weighted mean and functional dispersion for flower duration are larger in northwestern and southeastern China, while they are smaller in central China (Figure 1e,f). The highest woody vegetation NPP are observed in southern China, while central and northeastern China exhibit the lowest NPP (Figure S5a).

### 3.2 Functional diversity‐NPP relationship and its climatic sensitivity

**FIGURE 2:**
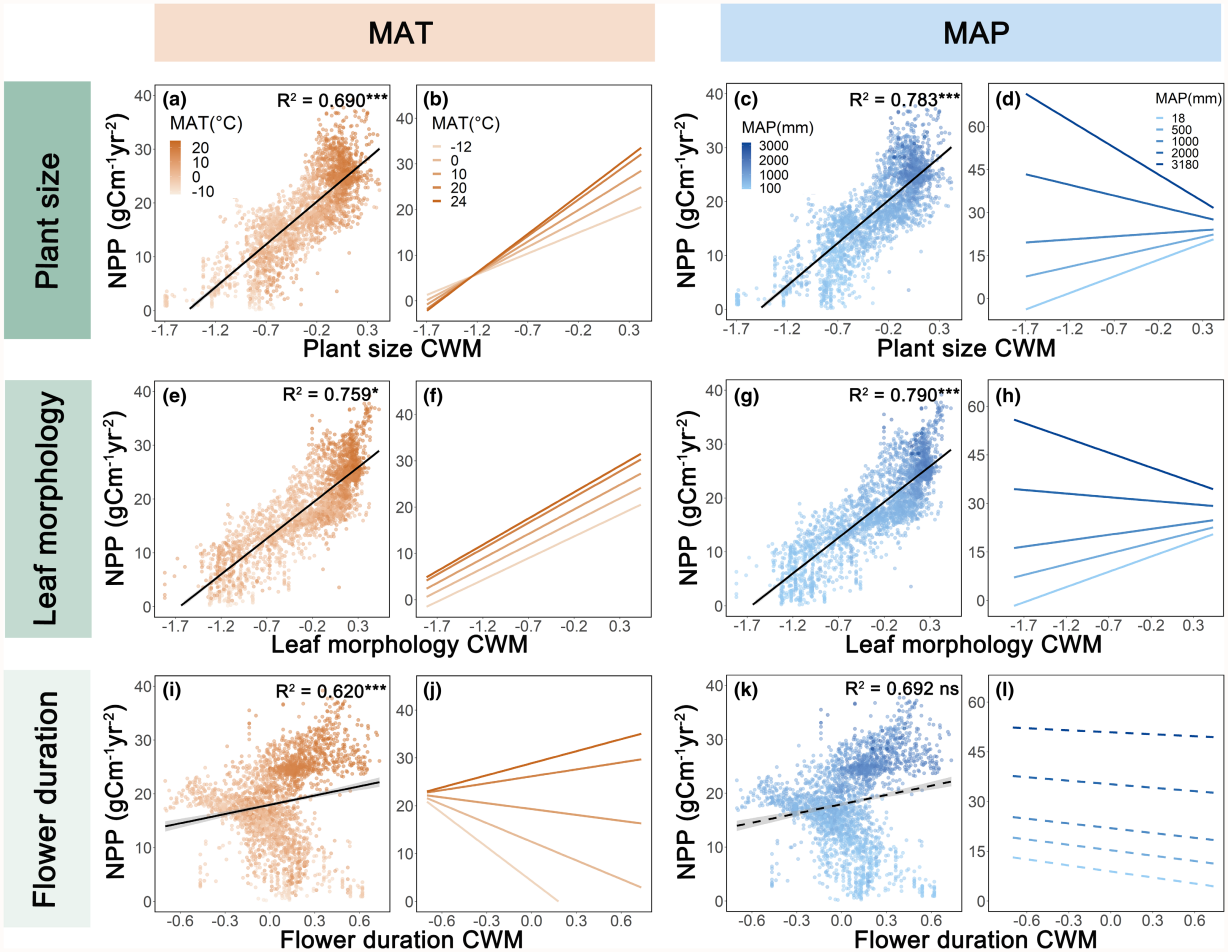
Linear regressions and predictions between community‐weighted mean and NPP under MAT and MAP. The community‐weighted mean of plant size (a), leaf morphology (e), flower duration (i) and NPP under MAT. The community‐weighted mean of plant size (c), leaf morphology (g), flower duration (k) and NPP under MAP. Black lines indicate the CWM–NPP relationships under MAT and MAP. The *R* ^2^ values were estimated with linear regression models, by using MAT (or MAP), CWM values, and their interaction as independent variables. The statistical significances (***p < .001; **p < .01; *p < .05) refer to interaction terms. (b), (f), (j) and (d), (h) and (l) were back‐transformed from linear regression models. Fitted lines are shown for CWM values covering the range from the grid with lowest to highest value. Dashed lines indicate non‐significant interactions. CWM, community‐weighted mean; MAP, mean annual precipitation (mm year^−1^); MAT, mean annual temperature (°C); NPP, woody vegetation productivity (g C m^−2^ year^−1^).

**FIGURE 3:**
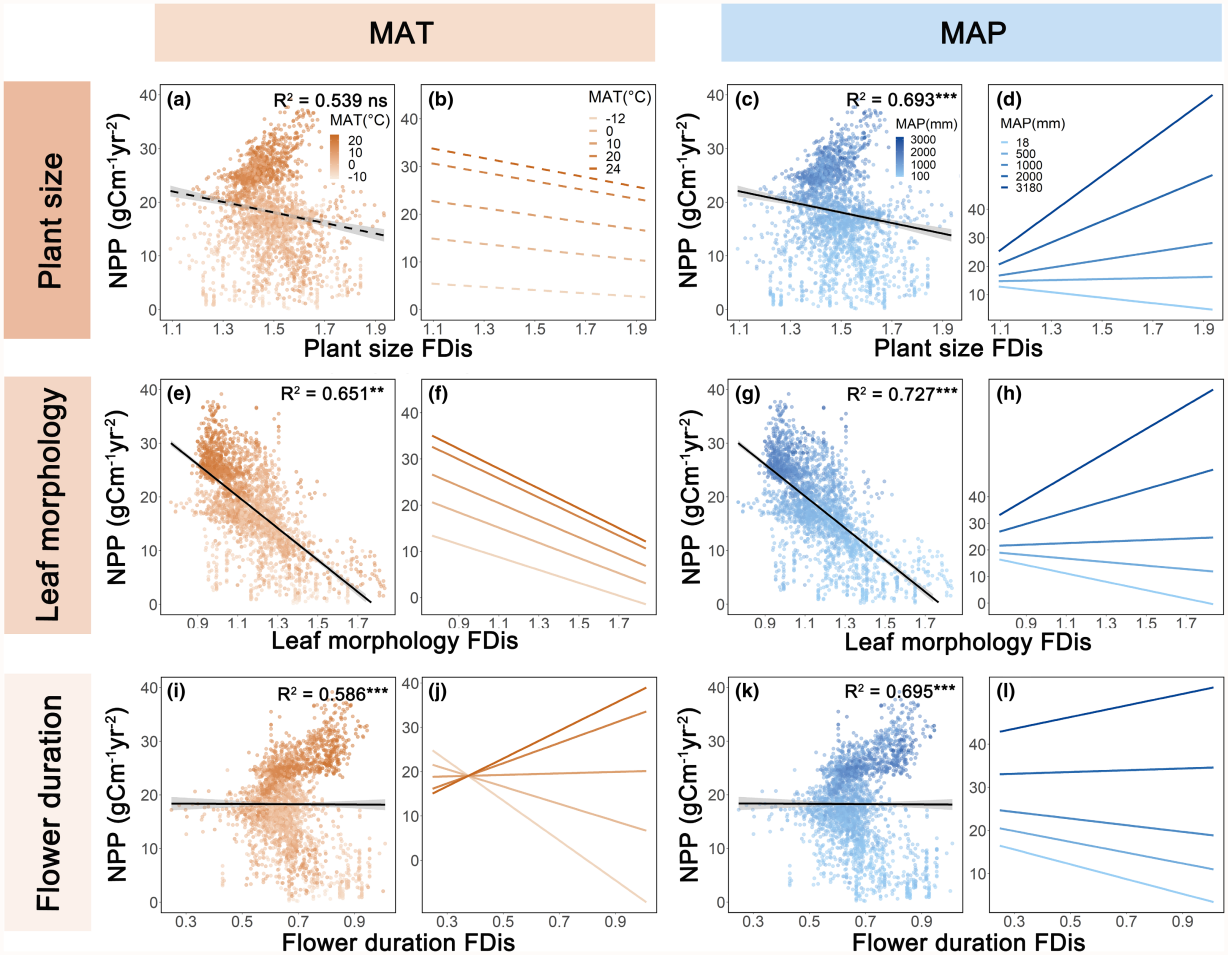
Linear regressions and predictions between functional dispersion and NPP under MAT and MAP. Functional dispersion of plant size (a), leaf morphology (e), flower duration (i) and NPP under MAT. Functional dispersion of plant size (c), leaf morphology (g), flower duration (k) and NPP under MAP. Black lines indicate the FDis–NPP relationships under MAT and MAP. The *R* ^2^ values were estimated with linear regression models, by using MAT (or MAP), FDis values, and their interaction as independent variables. The statistical significances (***p < .001; **p < .01; *p < .05) refer to interaction terms. (b), (f), (j) and (d), (h) and (l) were back‐transformed from linear regression models. Fitted lines are shown for FDis values covering the range from the grid with lowest to highest value. Dashed lines indicate non‐significant interactions. FDis, functional dispersion; MAP, mean annual precipitation (mm year^−1^); MAT, mean annual temperature (°C); NPP, woody vegetation productivity (g C m^−2^ year^−1^).

Significant interactions were observed between climatic conditions and functional diversity, suggesting that the relationship between functional diversity and NPP was sensitive to climate. Overall, the results indicated that the positive general relationships of both community‐weighted mean and functional dispersion with NPP increased in magnitude in warm and wetter climates, respectively (Figures 2 and 3; Tables S4, S5 and S6). In warmer regions, larger plant size, leaf morphology, and flower duration community‐weighted mean increased NPP compared to colder regions (Figure 2b,f,j). Similarly, larger plant size, leaf morphology, and flower duration functional dispersion were found to promote more NPP in wet regions than in dry regions (Figure 3d,h,l). Furthermore, the positive relationships of most community‐weighted mean/functional dispersion variables with NPP decreased as temperatures became less stable, but leaf morphology functional dispersion enhanced NPP more strongly under paleoclimatically unstable conditions than stable conditions (Figure S7h). These results remained significant even after correcting for spatial autocorrelation using SAR models (Figures S8; Tables S7, S8 and S9).

### 3.3 Climatic conditions affect NPP through functional diversity

**FIGURE 4:**
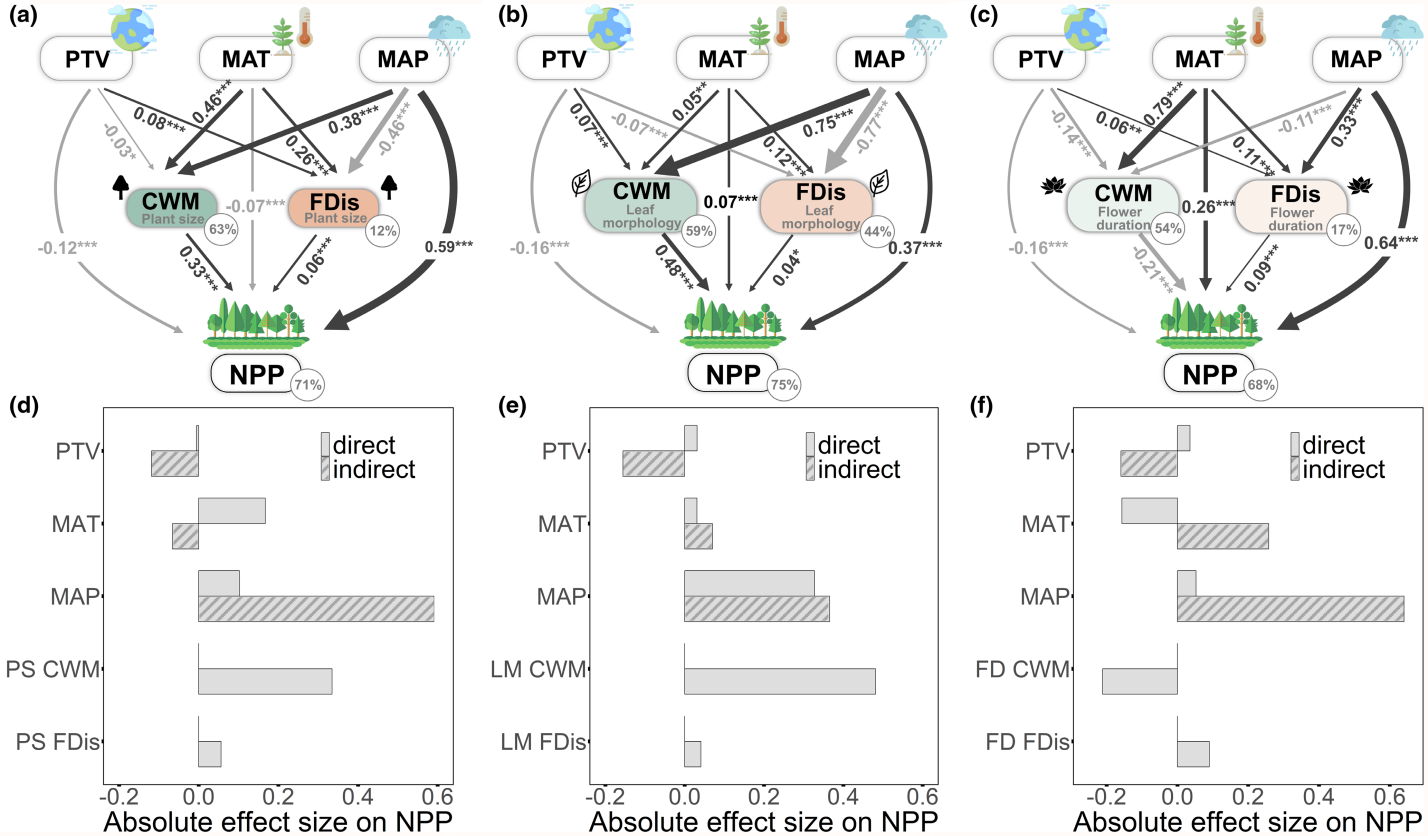
Path diagrams and path effects for climatic conditions and the CWM and FDis values of three axes of woody vegetation functional diversity and NPP across China. Plant size CWM and FDis (a), Leaf morphology CWM and FDis (b), Flower duration CWM and FDis (c). Arrows represent the hypothesized causal relationships between variables and arrow widths are proportional to the strength of relationships. The arrows are dark grey for positive correlation and light grey for negative correlation. Numbers next to the arrows are standardized path coefficients with associated statistical significance (***p < .001; **p < .01; *p < .05). The absolute effect size of climatic conditions and functional diversity on NPP obtain from the SEM model for Plant size CWM and FDis (d), Leaf morphology CWM and FDis (e), and Flower duration CWM and FDis (f). Effects are either direct (gray bar of each climatic condition and functional diversity affect NPP via direct path) or indirect via functional diversity (gray bar with slanted lines of each climatic condition, effects of climatic conditions on functional diversity, which in turn affect NPP). Absolute values are used to compare the effect size, which is calculated as the product of standardized path coefficients. The indirect effects of climatic conditions on NPP result from the summation of all pathways of indirect effects through CWM and FDis. CWM, community‐weighted mean; FD, flower duration; FDis, functional dispersion; LM, leaf morphology; MAT, mean annual temperature (°C); MAP, mean annual precipitation (mm year^−1^); NPP, woody vegetation productivity (g C m^−2^ year^−1^); PS, plant size; PTV, paleotemperature velocity (m year^−1^).

**FIGURE 5:**
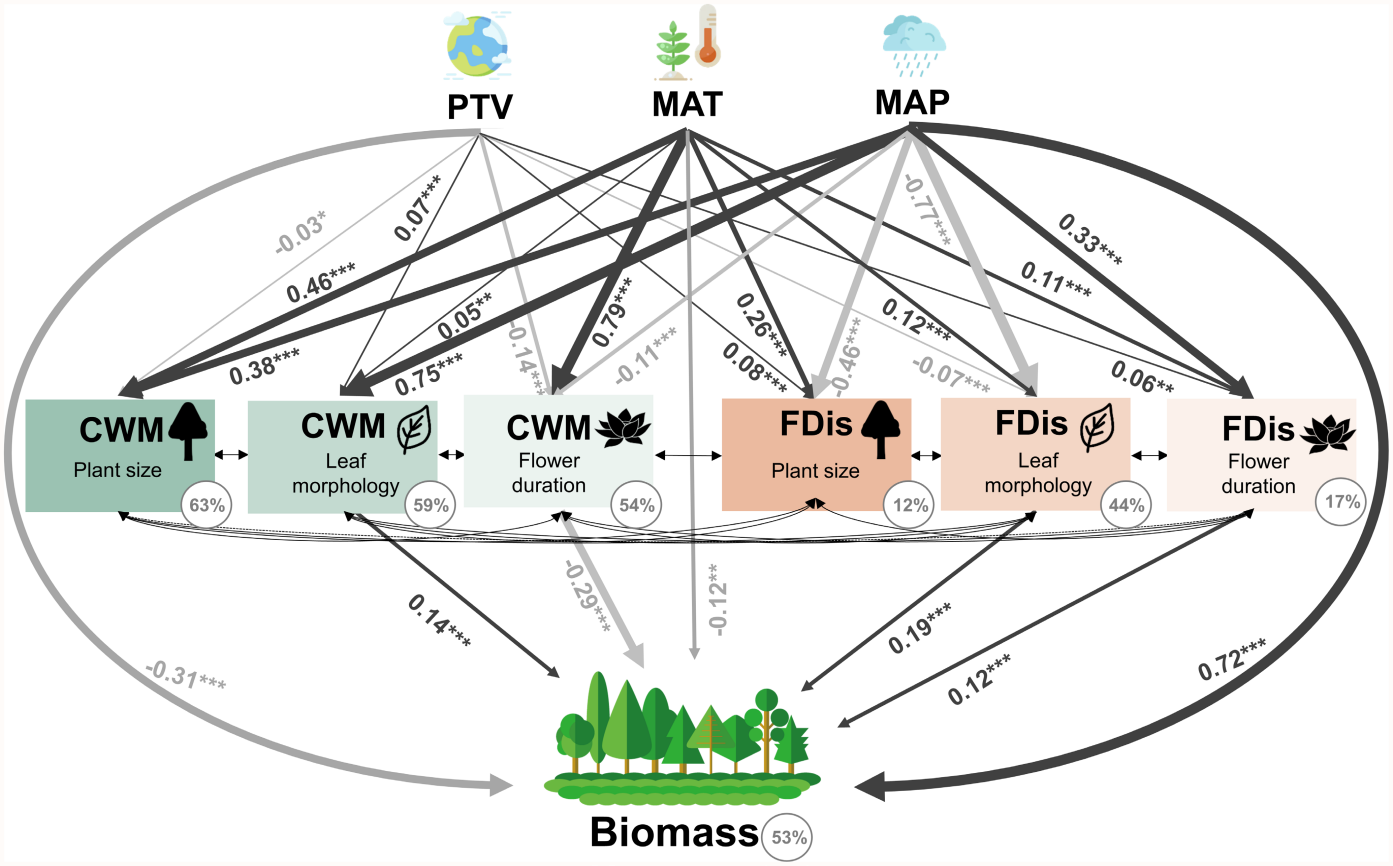
SEM model of direct and indirect effects of climatic conditions and FDs on the sum of forest aboveground biomass (Fishers' *C* = 10.41, df = 4, *p* = .034, *n* = 2340). Arrows represent the hypothesized causal relationships between variables and arrow widths are proportional to the strength of relationships. The arrows are dark grey for positive correlation and light grey for negative correlation. Numbers next to the arrows are standardized path coefficients with associated statistical significance (***p < .001; **p < .01; *p < .05). Biomass, forest aboveground biomass (Mg ha^−1^); CWM, community‐weighted mean; FDis, functional dispersion; MAP, mean annual precipitation (mm year^−1^); MAT, mean annual temperature (°C); PTV, paleotemperature velocity (m year^−1^).

NPP was found to be higher in regions with more stable paleoclimatic conditions and higher precipitation, indicating direct climatic effects (Figure 4a–c; Tables S10, S11 and S12). However, climatic conditions also exerted an indirect effect on NPP through their impact on functional diversity. In warmer and wetter regions, plant size or leaf morphology community‐weighted mean were larger (Figure 4a,b). Plant size functional dispersion in warm regions or flower duration functional dispersion in warm and wet regions were found to promote NPP (Figure 4a,c). In combination, climatic conditions and functional diversity explained approximately 70% of the total variation in NPP across China in models considering three trait groups, respectively. MAP was identified as the most related climatic condition to NPP, while mean plant size and leaf morphology were identified as the most related functional diversity indices (Figure 4d–f). A similar pattern was observed in the full model of three traits, where the indirect effects of climatic conditions on NPP were larger than the direct effects (Figure S9; Table S13). Furthermore, both direct and indirect effects of climatic conditions on AGB were identified, explaining 54% of the total variation in AGB (Figure 5; Table S14). Leaf morphology community‐weighted mean and functional dispersion as well as flower duration functional dispersion, were found to be the factors that most strongly promote AGB.

## 4 DISCUSSION

Maintaining diverse ecosystem functions such as biomass production and carbon capture is critical to sustaining ecosystem services that benefit human beings (Isbell et al., 2015). Consequently, there is an urgent to comprehend the roles of functional diversity as a driver of ecosystem functioning under various climatic conditions. In this study, we gathered functional traits from 9120 Chinese woody species and modeled the NPP of woody vegetation based on various aspects of functional diversity, particularly focusing on community‐weighted mean and functional dispersion of trait groups, across different climatic conditions in China. Consistent with the macroecological complementarity hypothesis (Burley et al., 2016; Chisholm et al., 2013), our findings indicate positive functional diversity–NPP relationships in Chinese woody vegetation that are sensitive to climate. Community‐weighted mean dimensions contribute more to the positive relationships in warm regions, while functional dispersion dimensions play a more significant role in wet regions. In regions characterized by a combination of warm and wet conditions, woody plant communities exhibiting both large plant size and leaf morphology community‐weighted mean and substantial flower duration functional dispersion were particularly effective in promoting high NPP. This suggests an increased importance of both aspects of functional diversity in warmer or wetter climates. These results enhance our understanding of the sensitivity of biodiversity–ecosystem functioning relationships in woody vegetation to spatial climatic variation and, by extension, to climate change over time, under different global‐change scenarios. Assuming ongoing warming trends, our findings suggest that the significance of maintaining high functional diversity and considering its interaction with climate will become even more critical in the future to ensure ecosystem functions and services provided by woody vegetation at a large spatial scale. This consideration should be incorporated into protection and restoration efforts, as well as in natural capital accounting for forest ecosystems.

### 4.1 Regularities of functional diversity distribution patterns

In southeastern China, where environmental conditions are favorable, species exhibit accelerated growth rates to acquire essential resources, such as light and water, resulting in an increase in both mean plant size and leaf morphology (Wang et al., 2019). Species coexisting in cold and dry regions demonstrate larger functional differences in plant size, leaf morphology, and flower duration compared to those in warm and wet regions. Our findings indicate that both the diversity and mean of flower duration are higher in tropical China, potentially influenced by varying offset dynamics of spring and summer‐flowering plants. Warm and wet regions have the ability to advance the flowering onset of spring‐flowering plants and delay it for summer‐flowering plants, thereby extending the flowering duration for the entire community (Li et al., 2021). The distribution pattern of woody vegetation species richness and its correlation with functional diversity (Figure S10) underscores the significance of the three‐dimensional functional dispersion in each community.

### 4.2 The relationships between functional diversity and NPP modulated by climate

The modification of functional diversity–NPP relationships by climatic conditions is evident through significant climatic conditions × functional diversity interactions for the three trait groups. In alignment with recent findings (Li et al., 2020; Wang et al., 2019), our results indicated that on average across various climatic conditions in China, the NPP of woody vegetation is predominantly influenced by species with large plant size and high leaf morphology (Figure 2a,e). This fits well with the common understanding that the mean tree height or mean leaf area contributes NPP due to the selection effect on species with specific traits affecting stand biomass (Šímová et al., 2019; Wang et al., 2019) or productivity (Li et al., 2020).

Communities with species exhibiting large plant size, leaf morphology, and flower duration are associated with high NPP, and this relationship becomes stronger in warm regions. However, when considering regions with diverse climatic conditions, the relationship between plant size community‐weighted mean and NPP becomes less important and the association between plant size and leaf morphology functional dispersion with NPP becomes more crucial in wet regions than large community‐weighted mean. This suggests that in wet climatic conditions, diverse tree communities may benefit more from niche complementarity, contradicting the stress‐gradient hypothesis but supporting findings that biodiversity effects increase with available biotope space (Dimitrakopoulos & Schmid, 2004). Moreover, the importance of flower duration community‐weighted mean and functional dispersion on productivity is weaker compared to the dimensions of plant size and leaf morphology. Flower duration is not strongly correlated with overall plant growth, making a relatively minor contribution to NPP. This indicates that under climatic conditions conducive to plant growth, such as in wet regions, the coexistence of species with different traits in woody plant communities promotes productivity through resource partitioning and facilitation, extending the results from plot‐scale experiments (Bongers et al., 2021; Huang et al., 2018) and observational studies (Liu et al., 2018) to larger spatial scales.

On one hand, greater leaf morphology functional dispersion may have improved ecosystem stability during geological times when paleoclimatic conditions were unstable (Garcia‐Palacios et al., 2018). On the other hand, species loss due to paleoclimatic instability may have reduced functional diversity and its positive relationships with NPP in less favorable climatic areas (Swenson et al., 2016). The results suggest that contrary to the initial hypothesis (H1), the positive functional dispersion (with regard to diversity of plant size and leaf morphology)–NPP relationships can be more pronounced under favorable climatic conditions than under stressful ones.

This observation aligns with experimental studies and the concept of larger biotope space availability in more beneficial environments, contributing to stronger positive relationships between diversity and productivity (Dimitrakopoulos & Schmid, 2004). In wet regions, a positive correlation between diversity and productivity is evident (Poorter et al., 2017). This relationship may be explained by the variation in traits relevant to resource partitioning among species promoted (Hisano et al., 2019), including partitioning of light between canopy and understory woody plants (Wright, 1992). The positive relationships between functional dispersion and NPP in wet regions illustrate the potential advantages of planting species with a diversity of plant sizes and leaf morphology in such regions. The functional differences between species in these regions might contribute more significantly to community productivity through resource partitioning, abiotic facilitation, or biotic feedbacks compared to other regions (Barry et al., 2019). Overall, our study highlights that the importance of relationships between functional diversity and NPP increases with higher temperatures and wetter climates. Consequently, restoration and reforestation strategies aimed at promoting NPP should carefully consider not just functional diversity, but also its interaction with climate in future environments.

### 4.3 Climatic conditions affect NPP indirectly via functional diversity

Metabolic scaling theory emphasizes aboveground biomass as a fundamental predictor of NPP (Enquist et al., 2017; Michaletz et al., 2014). Thus, it is conceivable that relationships between functional diversity and NPP mainly or even fully work via aboveground biomass at a large spatial scale under various climatic conditions. To simplify the model and reduce complexity, an alternative approach involves using aboveground biomass as the response variable in the same SEM model (Figure 5). Our findings support Michaletz's perspective, establishing a connection between biodiversity theory and metabolic scaling theory in explaining large‐scale variations in NPP influenced by climate. That biomass‐related NPP is ultimately driven by functional diversity, that is, must rely on the presence of a set of species in a community that can produce the maximum biomass under given local environmental constraints, provides a novel perspective on trait‐based ecosystem studies at macro‐ecological scales.

Our findings indicate that NPP is predominantly influenced by functional diversity, but the functional diversity–NPP relationship can be modified by climatic conditions, with precipitation exerting a more significant influence than temperature. Climatic conditions may influence NPP directly or indirectly, affecting functional diversity through processes such as species filtering from regional species pools (Svenning et al., 2010). In warmer regions, diversity, mean plant size, and leaf morphology play a crucial role in driving NPP. Larger mean plant size and leaf morphology enhance the growth rate and plant metabolic rate (Boisvenue & Running, 2006), making communities more competitive in acquiring biotope space and light. Under dry conditions, lower species diversity may lead to functional homogeneity, resulting in less positive functional diversity–NPP relationships among plant size, leaf morphology, and flower duration diversity (Aguirre‐Gutierrez et al., 2020). However, these positive functional diversity–NPP relationships tend to recover when the environmental conditions become wet again (Dimitrakopoulos & Schmid, 2004). The indirect effects of climatic conditions on NPP through functional diversity are more substantial than the direct effects align with our expectation that climatic conditions shape functional diversity and influence ecosystem functioning at a large spatial scale (Garcia‐Palacios et al., 2018). In restoration, reforestation actions, and natural capital accounting, promoting functional diversity will be increasingly important to enhance productivity and resilience in woody vegetation across China if the future climate becomes warmer and wetter.

### 4.4 Limitations and future direction

The plasticity of traits contributes to species' resilience and adaptability, thereby influencing overall ecosystem functions in response to varying climatic conditions (Violle et al., 2012). Our analysis employed species average functional trait values, which might limit our understanding of how species' functional traits adapt to diverse climatic conditions. Additionally, the use of presence/absence data to calculate components of functional diversity could potentially result in an underestimation of the contribution of dominant plant species to NPP (Li et al., 2020). However, the impact of abundance‐weighted measures of functional diversity on our results remains uncertain, as the relationships between climatic conditions and functional values can be strengthened (Violle et al., 2012) or weakened (Pakeman et al., 2008) by abundance‐weighted compared to unweighted functional diversity measures. Finally, based on observations in natural forests, our study identified the correlations between climatic conditions and functional diversity–NPP relationship. Moving forward, future studies should focus on evaluating the uncertainty associated with functional traits' plasticity and considering species abundance when estimating functional diversity and its relationships with NPP in a changing climate. Additionally, manipulative experiments can be conducted to investigate potential causal relationships between climatic conditions and functional diversity‐NPP, exploring how these relationships may vary under different climatic conditions.

## 5 CONCLUSIONS

In sum, our study establishes the presence of positive relationships between community‐weighted mean/functional dispersion of three major trait syndromes and NPP across a wide range of climatic conditions in China. This extends our understanding of biodiversity–ecosystem functioning relationships from plot‐scale experimental and observational studies to a larger spatial scale and enriches the verification of large‐scale productivity‐driving factors. We highlight the climatic sensitivity of functional diversity–NPP relationships. Assemblages characterized by large plant size and leaf morphology community‐weighted mean exhibit high NPP in warm regions, whereas woody vegetation with larger plant size and leaf morphology functional dispersion promotes NPP, especially in wet regions. Furthermore, the results suggest warm and wet conditions promote NPP indirectly through plant size and leaf morphology community‐weighted mean. Our findings underscore the sensitivity of large‐scale BEF relationships in woody vegetation to climate change, emphasizing the growing importance of plant traits for woody vegetation NPP with rising temperatures and decreasing water limitation. We emphasize the importance of considering potential changes in local climatic conditions in restoration, reforestation actions, and natural capital accounting. This approach optimizes the value of community functional diversity and considers its interaction with climate, thus promoting productivity and related ecosystem services.

## AUTHOR CONTRIBUTIONS

**Haoru Yan:** Data curation (lead); formal analysis (lead); methodology (equal); writing – original draft (lead). **Bernhard Schmid:** Funding acquisition (equal); methodology (equal); writing – review and editing (equal). **Wubing Xu:** Data curation (equal); methodology (equal); writing – review and editing (equal). **Franca J. Bongers:** Formal analysis (equal); visualization (equal); writing – review and editing (equal). **Guoke Chen:** Data curation (equal); writing – review and editing (equal). **Ting Tang:** Data curation (equal); formal analysis (equal); writing – review and editing (equal). **Zhiheng Wang:** Writing – review and editing (equal). **Jens‐Christian Svenning:** Formal analysis (equal); funding acquisition (equal); writing – review and editing (equal). **Keping Ma:** Conceptualization (equal); data curation (equal); funding acquisition (equal); supervision (equal); writing – review and editing (equal). **Xiaojuan Liu:** Conceptualization (lead); data curation (lead); formal analysis (lead); funding acquisition (lead); supervision (lead); writing – review and editing (lead).

## CONFLICT OF INTEREST STATEMENT

The authors declare no competing interest.

## Supporting information


Appendix S1

## Data Availability

The data that support the findings of this study are available on datadryad.org (https://doi.org/10.5061/dryad.4f4qrfjg8).
