# Supplementary material for: The functional diversity–productivity relationship of woody plants is climatically sensitive
Source: Ecol Evol. 2024 May 2;14(5):e11364. doi: 10.1002/ece3.11364 (PMC11063782; doi:10.1002/ece3.11364)
Supplement: Supplementary file 1 — Appendix S1 [file ECE3-14-e11364-s001.docx]

**Supplementary Information for**

The functional diversity–productivity relationship of woody plants is climatically sensitive

Haoru Yan, Bernhard Schmid, Wubing Xu, Franca J. Bongers, Guoke Chen, Ting Tang, Zhiheng Wang, Jens-Christian Svenning, Keping Ma, Xiaojuan Liu^*^

*Corresponding Author: Xiaojuan Liu, liuxiaojuan06@ibcas.ac.cn

**This file includes:**

Figures S1 to S10

Tables S1 to S14


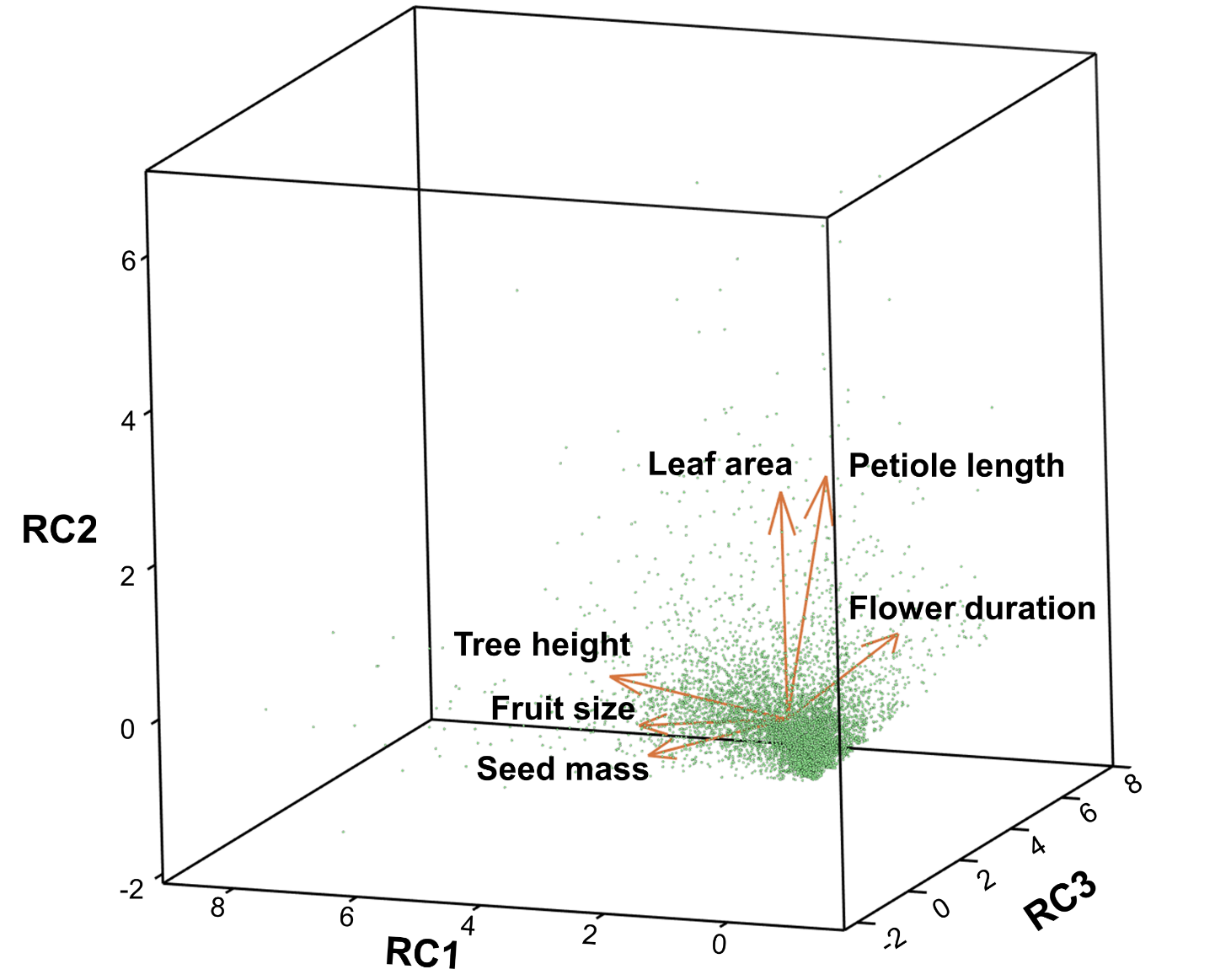


**Figure S1** Varimax rotation principal component analysis (PCA) plot for the original six functional traits.


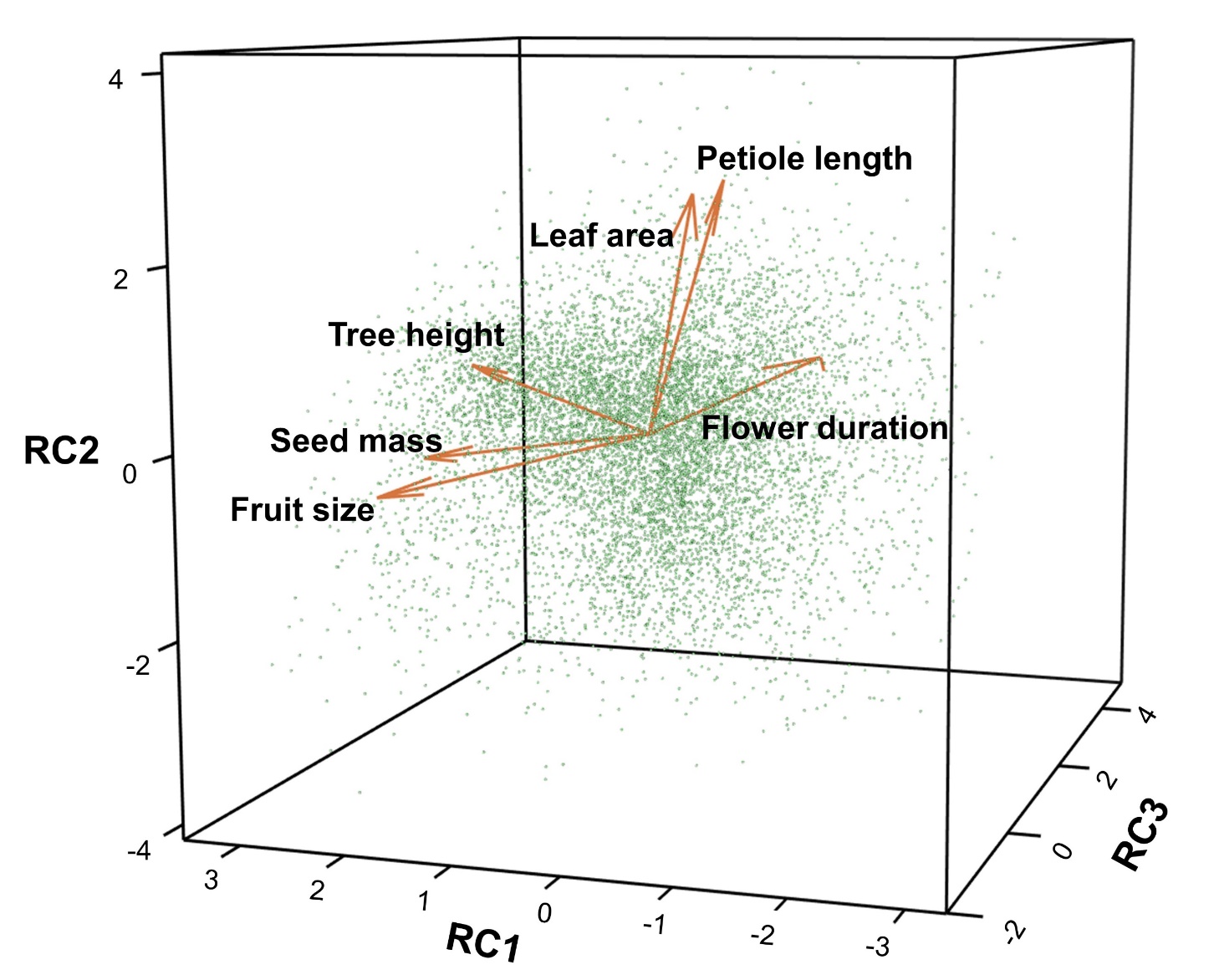


**Figure S2** Varimax rotation principal component analysis (PCA) plot for the filled six functional traits.


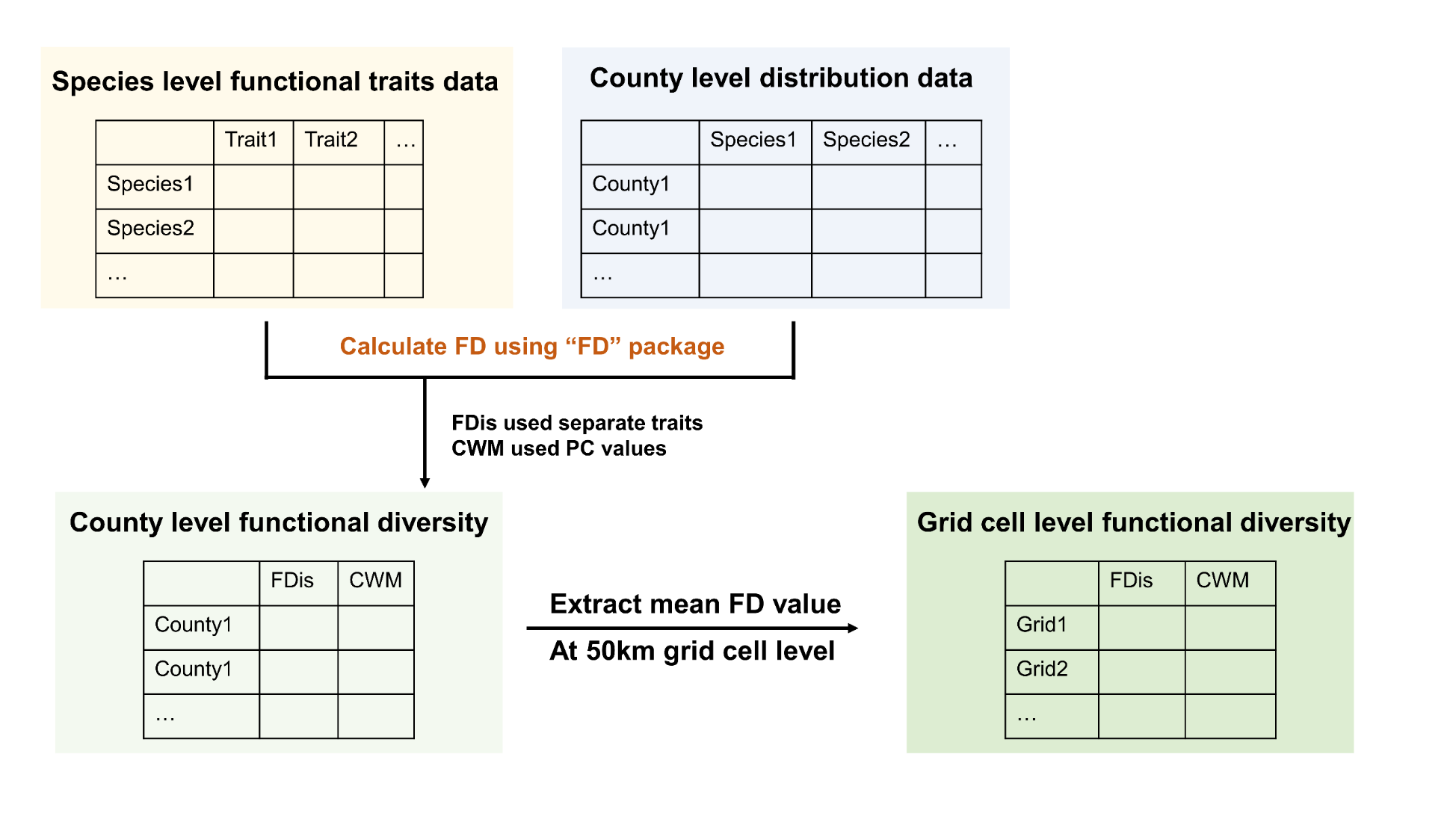


**Figure S3** The calculation process of the function diversity at the grid cell level.


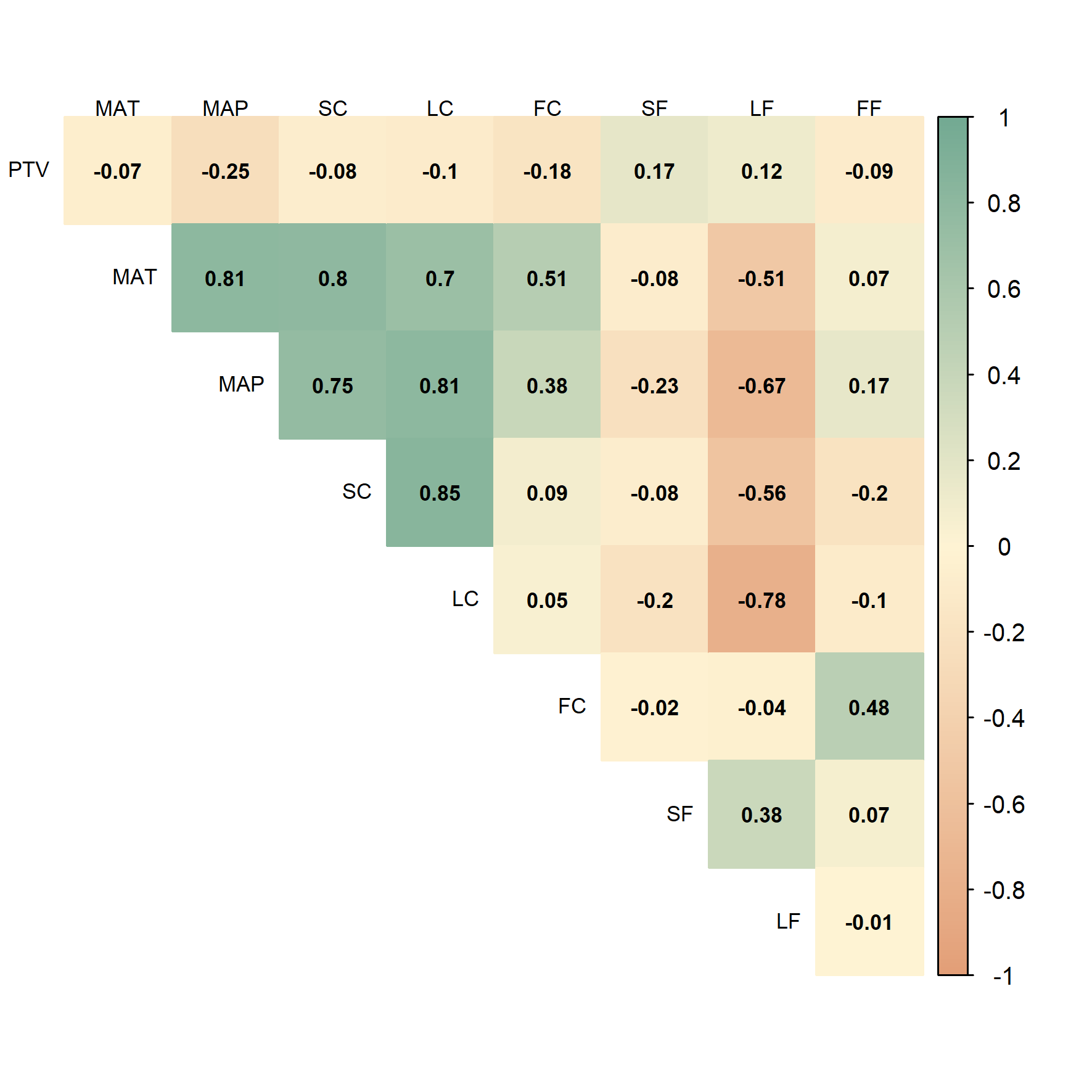


**Figure S4** Correlations of climatic conditions and functional diversity. PTV, paleo temperature velocity (m yr^-1^); MAT, mean annual temperature (°C); MAP, mean annual precipitation (mm yr^-1^); SC, plant size CWM; LC, leaf morphology CWM; FC, flower duration CWM; SF, plant size FDis; LF, leaf morphology FDis; FF, flower duration FDis; NPP, woody vegetation productivity (g C m^-2^ yr^-1^). CWM, community-weighted mean; FDis, functional dispersion.


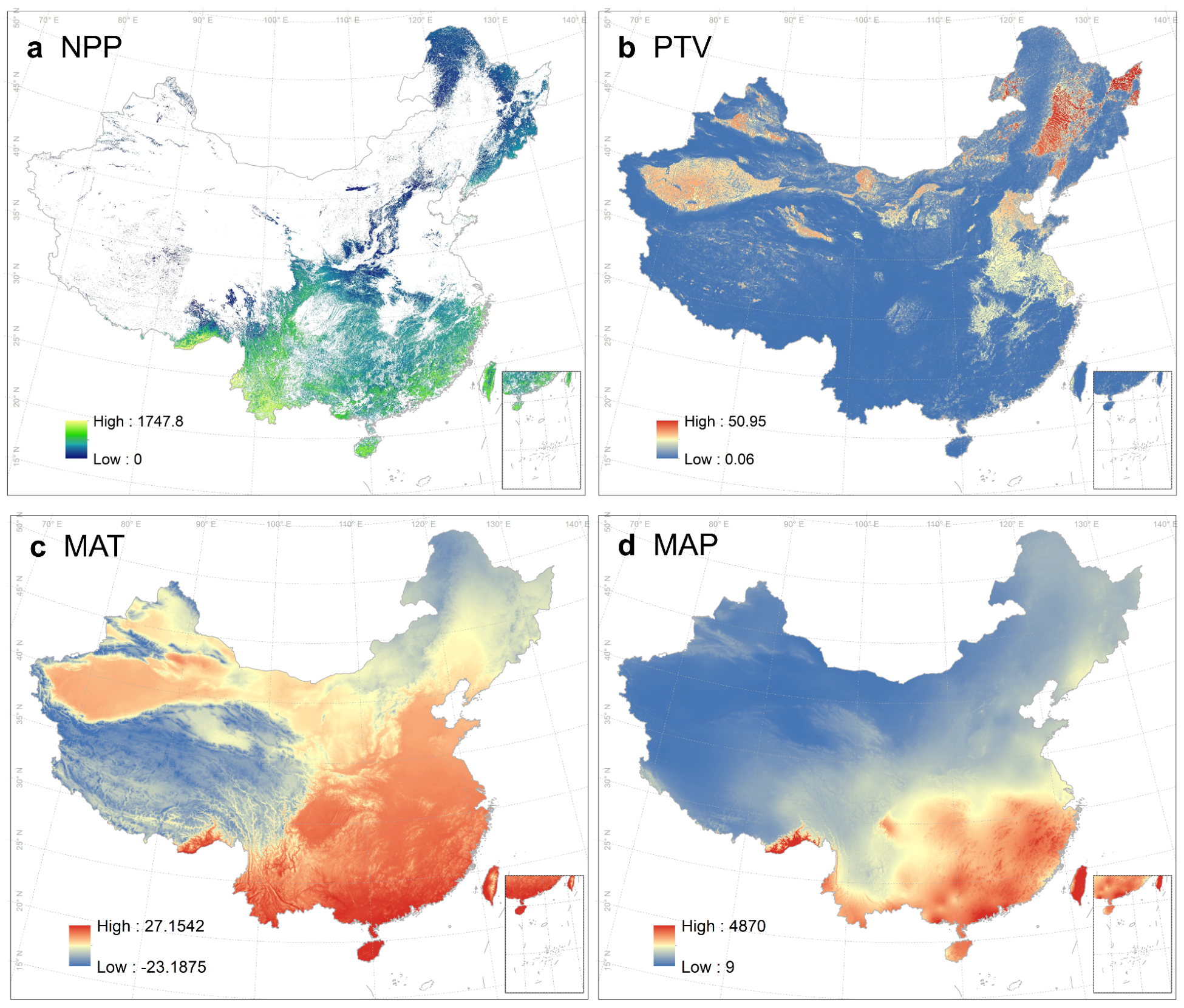


**Figure S5** Woody vegetation productivity and the three selected climatic variables: NPP (a), PTV (b), MAT (c), MAP (d). NPP, woody vegetation productivity (g C m^-2^ yr^-1^); PTV, paleo temperature velocity (m yr^-1^); MAT, mean annual temperature (°C); MAP, mean annual precipitation (mm yr^-1^).


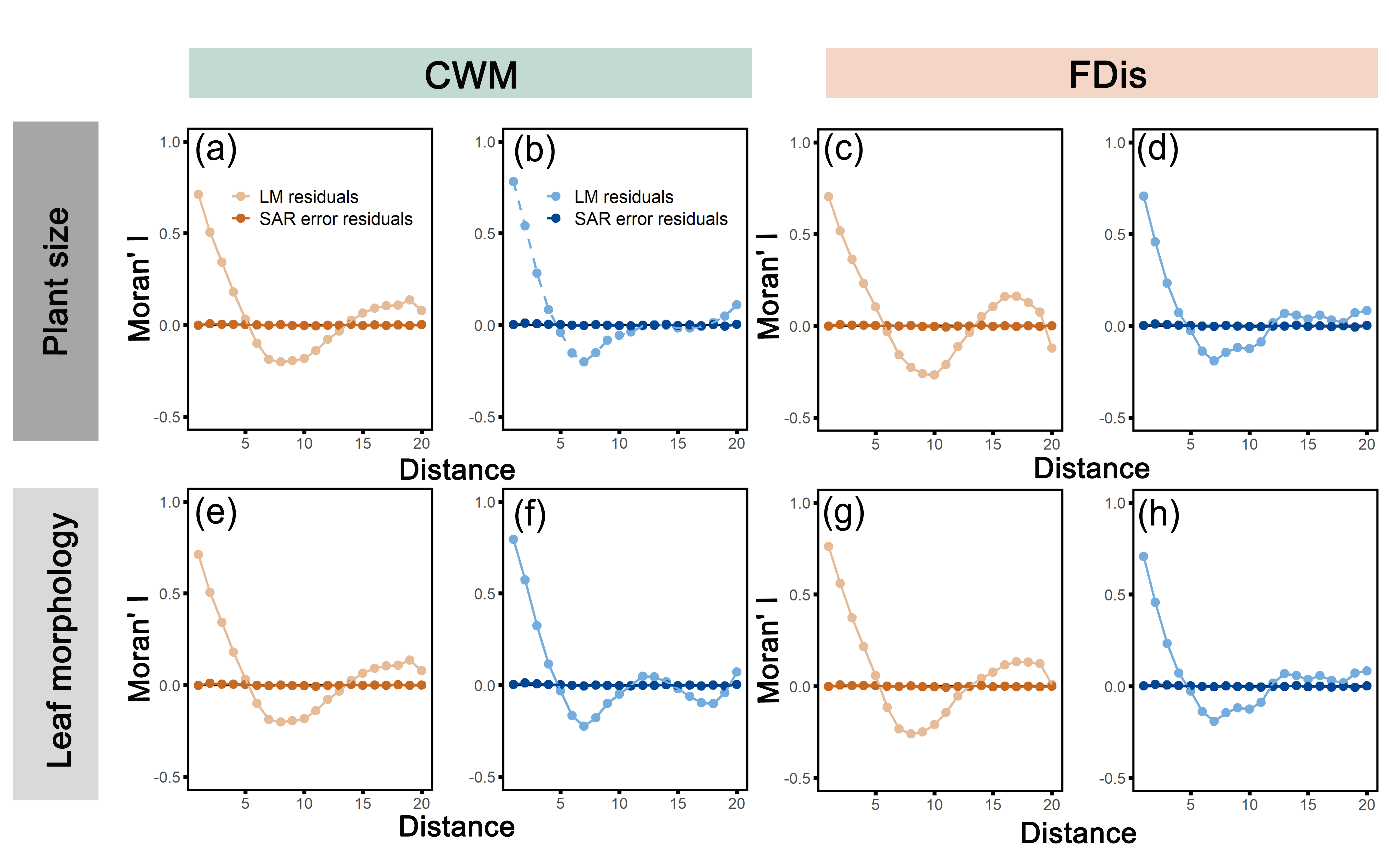


**Figure S6** Spatial autocorrelation structures (Moran’s *I*) for observed values of range size and residuals of range size from multiple linear regression (LM) and spatial simultaneous autoregressive error model (SAR) of functional diversity. The distance of plant size CWM (**a**), leaf morphology CWM (**e**) and NPP under MAT. The distance of plant size CWM (**b**), leaf morphology CWM (**f**) and NPP under MAP. The distance of plant size FDis (**c**), leaf morphology FDis (**g**) and NPP under MAT. The distance of plant size FDis (**d**), leaf morphology FDis (**h**) and NPP under MAP. CWM, community-weighted mean; FDis, functional dispersion; NPP, woody vegetation productivity (g C m^-2^ yr^-1^); MAT, mean annual temperature (°C); MAP, mean annual precipitation (mm yr^-1^).


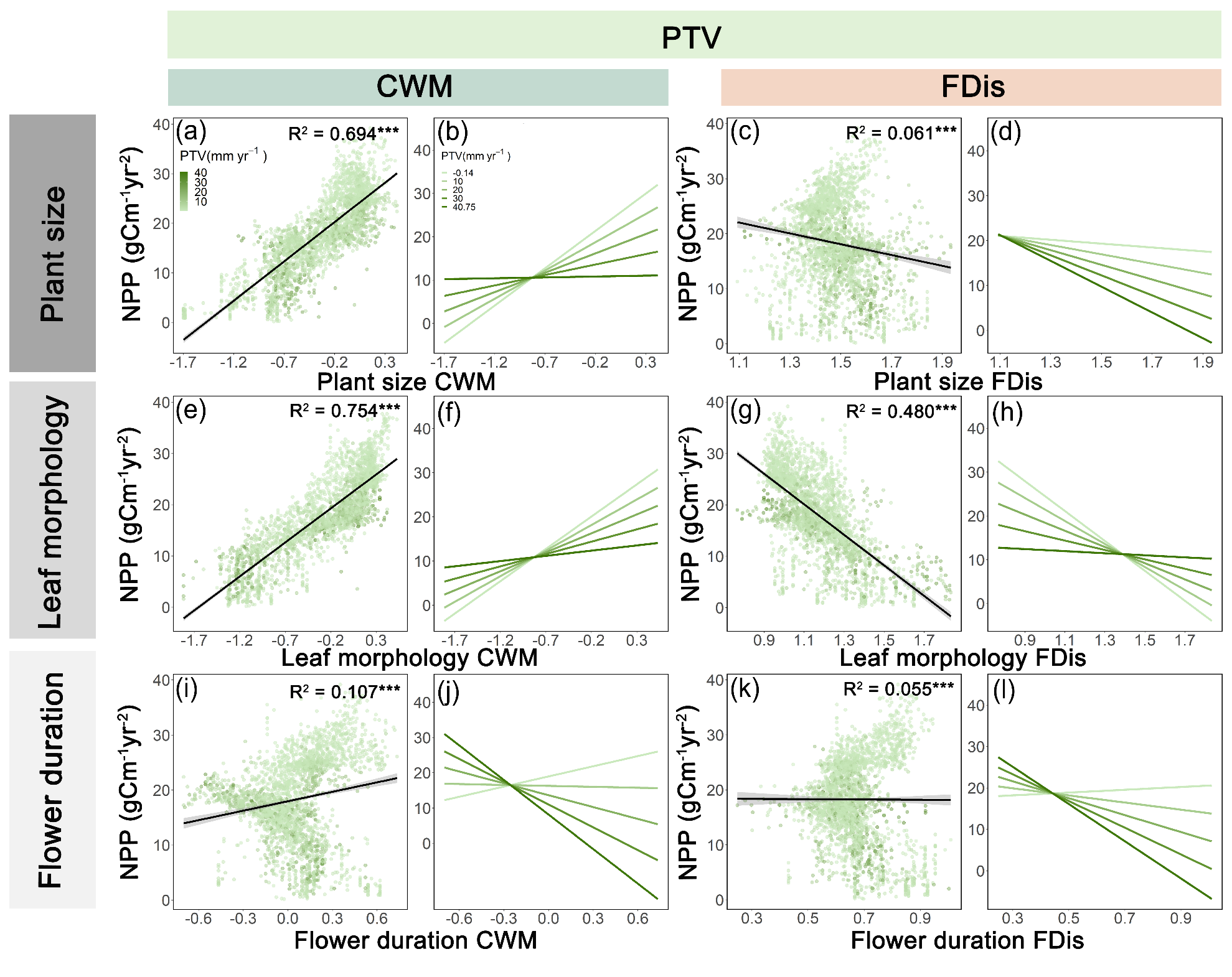


**Figure S7** Linear regressions and predictions between community-weighted mean or functional dispersion and NPP under PTV. Community-weighted mean of plant size (**a**), leaf morphology (**e**), flower duration (**i**) and NPP under PTV. Functional dispersion of plant size (**c**), leaf morphology (**g**), flower duration (**k**) and NPP under PTV. Black lines indicate the CWM-NPP and FDis-NPP relationships under PTV. The R^2^ values were estimated with linear regression models, by using PTV, CWM values or FDis values, and their interaction as independent variables. The statistical significances (***P < 0.001; **P < 0.01; *P < 0.05) were from interaction terms. (**b**), (**f**), (**j**) and (**d**), (**h**), (**l**) were back-transformed from the above linear regression model. Fitted line are shown for CWM or FDis values covering the range from the grid with lowest to highest value. CWM, community-weighted mean; FDis, functional dispersion; NPP, woody vegetation productivity (g C m^-2^ yr^-1^); PTV, paleo temperature velocity (m yr^-1^).

**
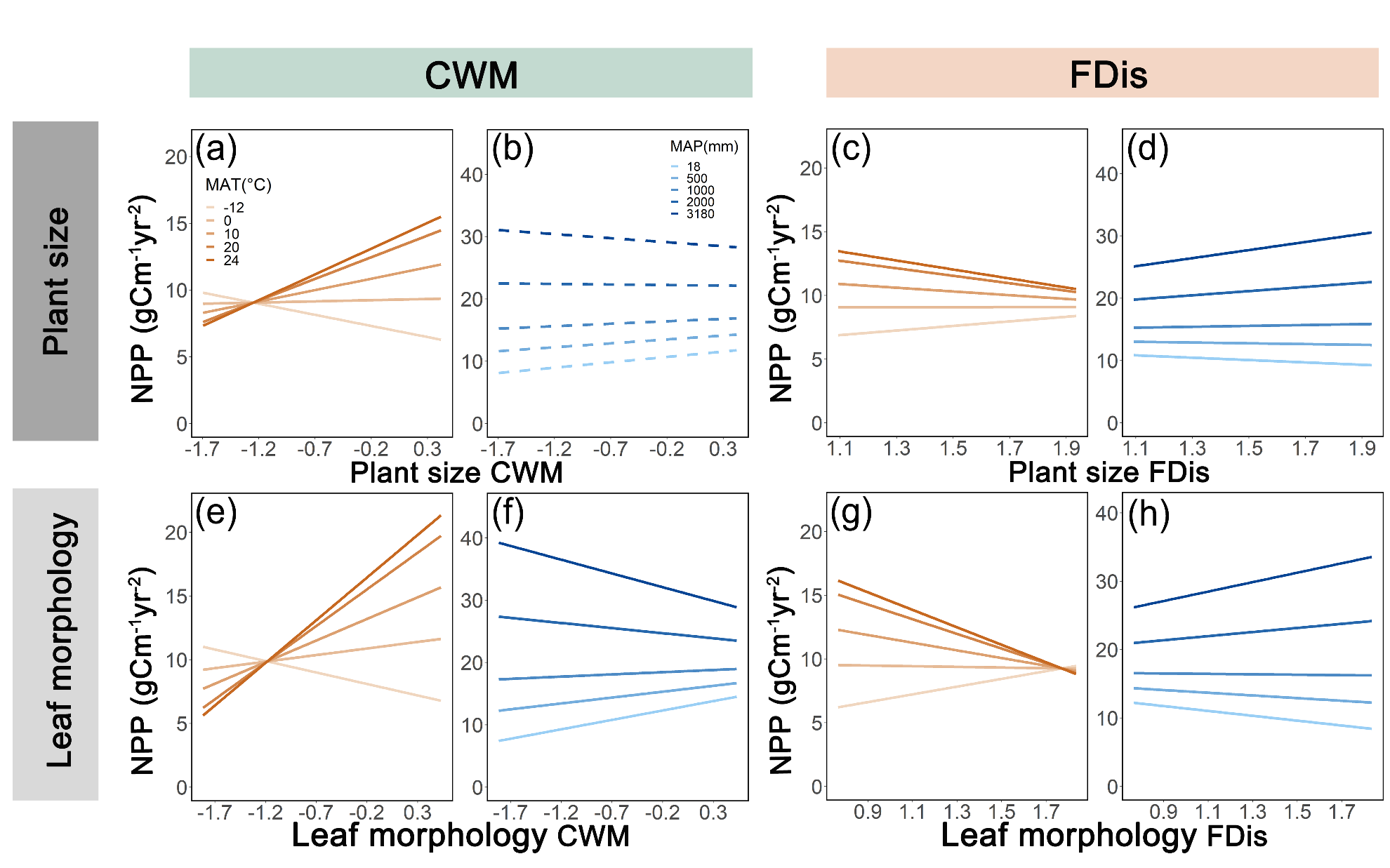
**

**Figure S8** SAR model predictions between community-weighted mean or functional dispersion and NPP under MAT and MAP. Community-weighted mean of plant size (**a**), leaf morphology (**e**) and NPP under MAT. Community-weighted mean of plant size (**b**), leaf morphology (**f**) and NPP under MAP. Functional dispersion of plant size (**c**), leaf morphology (**g**) and NPP under MAT. Functional dispersion of plant size (**d**), leaf morphology (**h**) and NPP under MAP. Fitted line are shown for CWM or FDis values covering the range from the grid with lowest to highest value. Dashed lines indicate the interaction relationships are non-significant. CWM, community-weighted mean; FDis, functional dispersion; NPP, woody vegetation productivity (g C m^-2^ yr^-1^); MAT, mean annual temperature (°C); MAP, mean annual precipitation (mm yr^-1^).


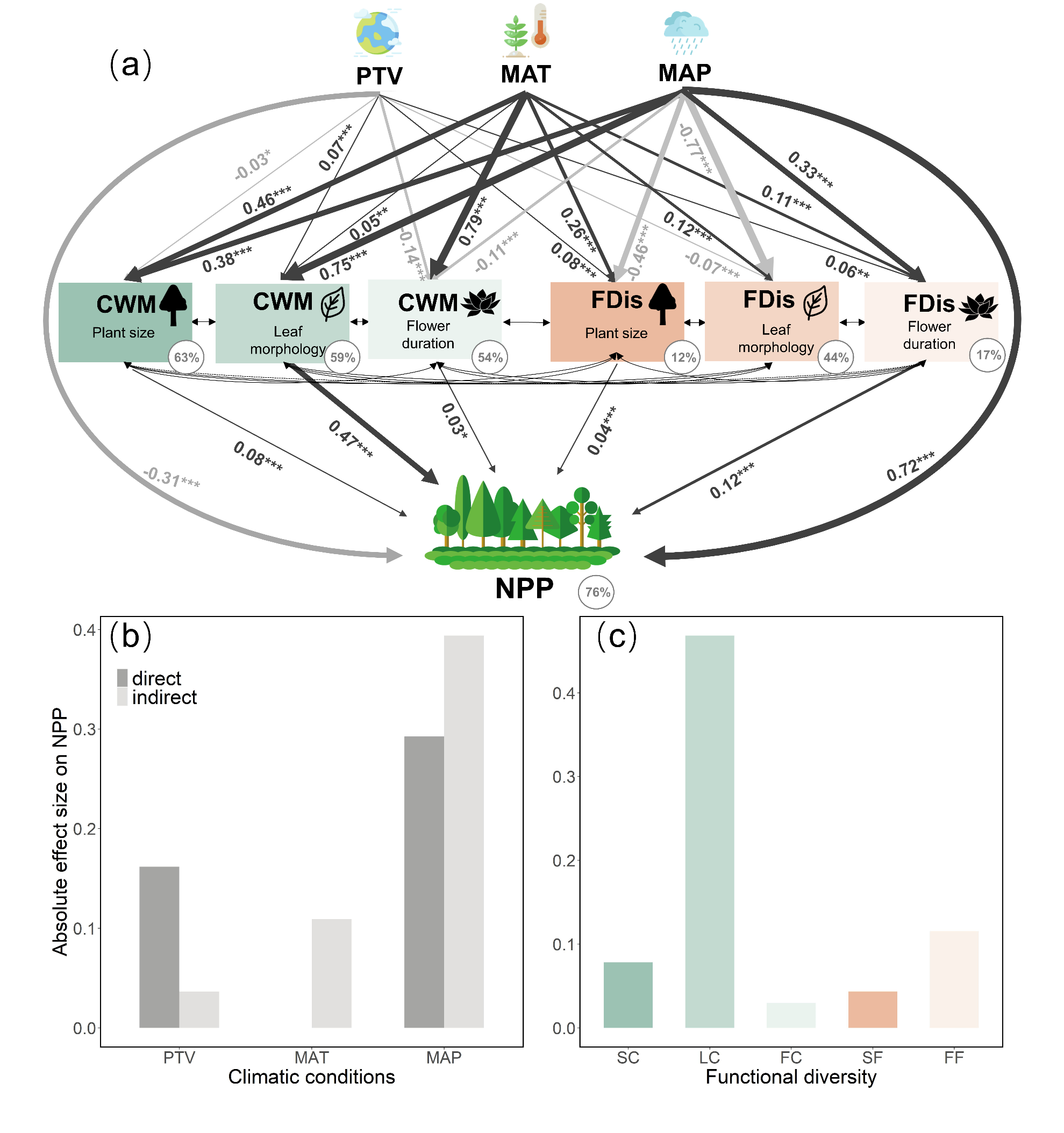


**Figure S9** Relationships between climatic conditions and the CWM and FDis values of three axes of woody vegetation functional diversity and NPP across China. **a** SEM model of direct and indirect effects of climatic conditions and FDs on NPP (Fishers’ C= 3.913, d.f.=4, P=0.418, n=2732). Arrows represent the hypothesized causal relationships between variables and arrow widths are proportional to the strength of relationships. The arrows are dark grey for positive correlation and light grey for negative correlation. Numbers next to the arrows are standardized path coefficients with associated statistical significance (***P < 0.001; **P < 0.01; *P < 0.05). The absolute effect size of climatic conditions (**b**) and functional diversity (**c**) on NPP. **b** Bar plot of direct (dark grey) and indirect (light grey) effects of climatic conditions on NPP obtained from the SEM model. Effects are either direct (dark grey bar of each climatic condition, connecting NPP with climatic conditions via direct path) or indirect via functional diversity (light grey bar of each climatic condition, effects of climatic conditions on functional diversity, which in turn affected NPP). Absolute values were used to compare the effect size, which was calculated as the product of standardized path coefficients. Components connecting each climatic condition with NPP were summed over the individual predictors for indirect effects. **c** The direct effects of CWM (green) and FDis (orange) on NPP. SC, plant size CWM; LC, leaf morphology CWM; FC, flower duration CWM; SF, plant size FDis; FF, flower duration FDis. Absolute values were used to compare the effect size. CWM, community-weighted mean; FDis, functional dispersion; NPP, woody vegetation productivity (g C m^-2^ yr^-1^); PTV, paleo temperature velocity (m yr^-1^); MAT, mean annual temperature (°C); MAP, mean annual precipitation (mm yr^-1^).


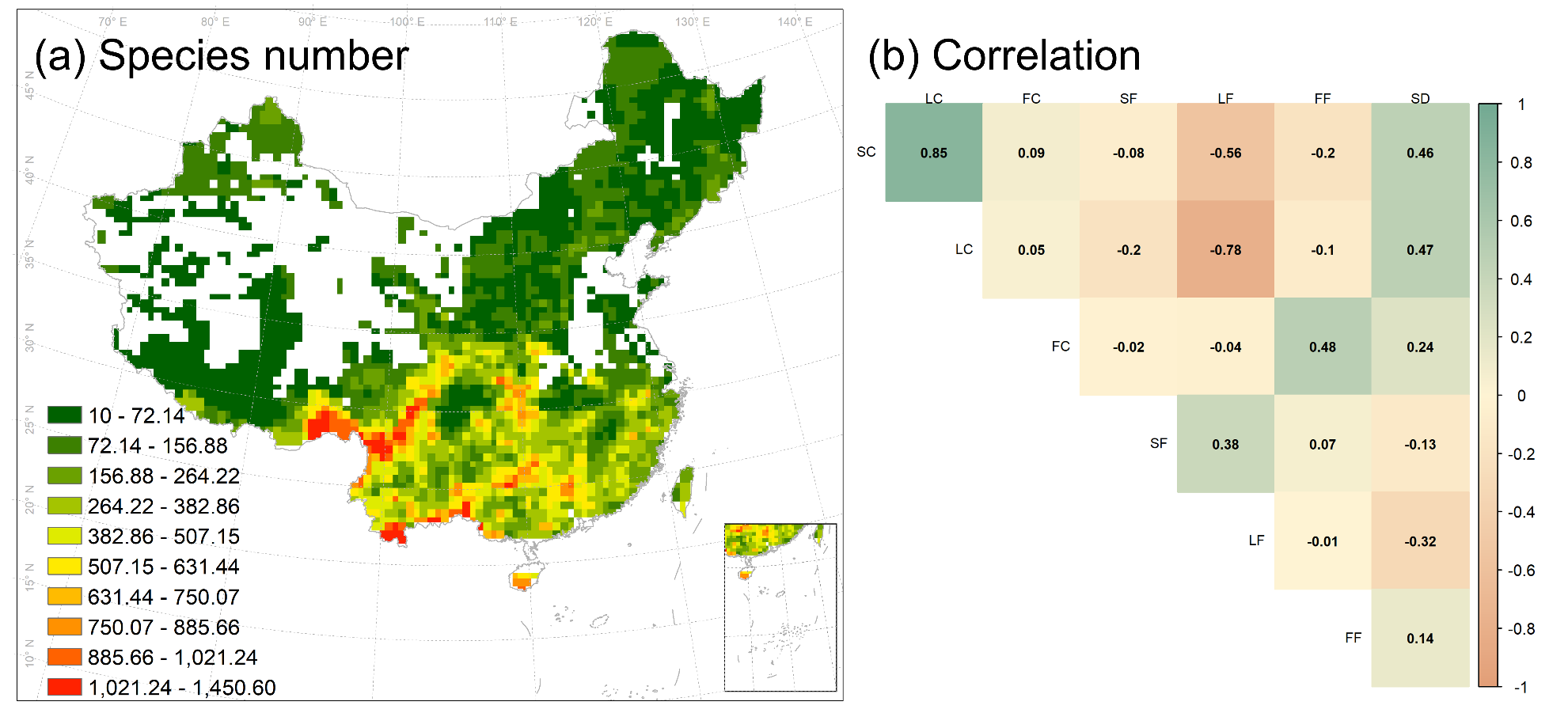


**Figure S10** The distribution pattern of species number in China and the correlations of functional diversity and species number. SC, plant size CWM; LC, leaf morphology CWM; FC, flower duration CWM; SF, plant size FDis; LF, leaf morphology FDis; FF, flower duration FDis; SD, species number. CWM, community-weighted mean; FDis, functional dispersion.

**Table S1** Number of species with functional traits values before and after genus mean value filling for total 9120 species.

| **Traits** | **Before value filling** | **After value filling** | **After value filling percentage** |
| --- | --- | --- | --- |
| Tree height | 7431 | 9093 | 99.7% |
| Flower duration | 7297 | 9027 | 99.0% |
| Leaf area | 8224 | 9018 | 98.9% |
| Fruit size | 7235 | 8958 | 98.2% |
| Petiole length | 6810 | 8618 | 94.5% |
| Seed mass | 2222 | 7982 | 87.5% |

**Table S2** Results of the varimax rotated Principal Component Analysis (PCA) of original traits at species level. The first three axes were selected, which together explained 62% of the variance.

|  | **RC1** | **RC2** | **RC3** | **h2** | **u2** |
| --- | --- | --- | --- | --- | --- |
| Fruit size | 0.70 | 0.03 | 0.09 | 0.50 | 0.50 |
| Seed mass | 0.65 | -0.08 | 0.10 | 0.44 | 0.56 |
| Tree height | 0.65 | 0.27 | -0.28 | 0.58 | 0.42 |
| Petiole length | -0.07 | 0.80 | 0.00 | 0.64 | 0.36 |
| Leaf area | 0.15 | 0.76 | 0.05 | 0.60 | 0.40 |
| Flower duration | 0.06 | 0.06 | 0.96 | 0.93 | 0.07 |
|  |  |  |  |  |  |
| *Proportion explained variance* | 0.23 | 0.22 | 0.17 |  |  |
| *Cumulative proportion explained variance* | 0.23 | 0.45 | 0.62 |  |  |

**Table S3** Results of the varimax rotated Principal Component Analysis (PCA) of filled traits at species level. The first three axes were selected, which together explained 75% of the variance.

|  | **RC1** | **RC2** | **RC3** | **h2** | **u2** |
| --- | --- | --- | --- | --- | --- |
| Fruit size | 0.83 | 0.01 | -0.04 | 0.68 | 0.32 |
| Seed mass | 0.80 | 0.10 | 0.66 | 0.66 | 0.34 |
| Tree height | 0.62 | 0.43 | -0.09 | 0.58 | 0.42 |
| Petiole length | 0.03 | 0.87 | -0.06 | 0.77 | 0.23 |
| Leaf area | 0.20 | 0.86 | 0.09 | 0.79 | 0.21 |
| Flower duration | 0.00 | 0.01 | 0.99 | 0.99 | 0.01 |
|  |  |  |  |  |  |
| *Proportion explained variance* | 0.29 | 0.28 | 0.17 |  |  |
| *Cumulative proportion explained variance* | 0.29 | 0.58 | 0.75 |  |  |

**Table S4** Linear model ANOVA table for the interactive effect of functional diversity and mean annual temperature (MAT) on NPP. P ≤ 0.05 are in boldface.

|  | **Factor** | **DF** | **Sumsq** | **F value** | ***P* value** |
| --- | --- | --- | --- | --- | --- |
| MATCWM1 | CWM1 | 1 | 111770 | 5819.4 | **<0.001** |
|  | MAT | 1 | 3880 | 202 | **<0.001** |
|  | CWM1:MAT | 1 | 919 | 47.9 | **<0.001** |
| MATCWM2 | CWM2 | 1 | 121933 | 8173.5 | **<0.001** |
|  | MAT | 1 | 6237 | 418.08 | **<0.001** |
|  | CWM2:MAT | 1 | 97 | 6.52 | **0.011** |
| MATCWM3 | CWM3 | 1 | 5232 | 222 | **<0.001** |
|  | MAT | 1 | 91818 | 3897 | **<0.001** |
|  | CWM3:MAT | 1 | 7645 | 324 | **<0.001** |
| MATFD1 | FD1 | 1 | 3626 | 127.13 | **<0.001** |
|  | MAT | 1 | 87454 | 3066.15 | **<0.001** |
|  | FD1:MAT | 1 | 75 | 2.62 | 0.11 |
| MATFD2 | FD2 | 1 | 74932 | 3464.6 | **<0.001** |
|  | MAT | 1 | 34812 | 1609.6 | **<0.001** |
|  | FD2:MAT | 1 | 219 | 10.2 | **0.002** |
| MATFD3 | FD3 | 1 | 2 | 0.08 | 0.78 |
|  | MAT | 1 | 90118 | 3513.72 | **<0.001** |
|  | FD3:MAT | 1 | 8878 | 346.17 | **<0.001** |

**Table S5** Linear model ANOVA table for the interactive effect of functional diversity and mean annual precipitation (MAP) on NPP. P ≤ 0.05 are in boldface.

|  | **Factor** | **DF** | **Sumsq** | **F value** | ***P* value** |
| --- | --- | --- | --- | --- | --- |
| MAPCWM1 | CWM1 | 1 | 111770 | 8317 | **<0.001** |
|  | MAP | 1 | 17182 | 1279 | **<0.001** |
|  | CWM1:MAP | 1 | 3351 | 249 | **<0.001** |
| MAPCWM2 | CWM2 | 1 | 121933 | 9356 | **<0.001** |
|  | MAP | 1 | 9779 | 750 | **<0.001** |
|  | CWM2:MAP | 1 | 1699 | 130 | **<0.001** |
| MAPCWM3 | CWM3 | 1 | 5232 | 274.59 | **<0.001** |
|  | MAP | 1 | 111707 | 5862.4 | **<0.001** |
|  | CWM3:MAP | 1 | 44 | 2.32 | 0.130 |
| MAPFD1 | FD1 | 1 | 3626 | 191 | **<0.001** |
|  | MAP | 1 | 109995 | 5784 | **<0.001** |
|  | FD1:MAP | 1 | 3467 | 182 | **<0.001** |
| MAPFD2 | FD2 | 1 | 74932 | 4431 | **<0.001** |
|  | MAP | 1 | 42751 | 2528 | **<0.001** |
|  | FD2:MAP | 1 | 5151 | 305 | **<0.001** |
| MAPFD3 | FD3 | 1 | 2 | 0.11 | 0.74 |
|  | MAP | 1 | 116787 | 6189.1 | **<0.001** |
|  | FD3:MAP | 1 | 699 | 37.06 | **<0.001** |

**Table S6** Linear model ANOVA table for the interactive effect of functional diversity and paleo-temperature velocity (PTV) on NPP. P ≤ 0.05 are in boldface.

|  | **Factor** | **DF** | **Sumsq** | **F value** | ***P* value** |
| --- | --- | --- | --- | --- | --- |
| PTVCWM1 | CWM1 | 1 | 111770 | 5904.1 | **<0.001** |
|  | PTV | 1 | 3685 | 194.7 | **<0.001** |
|  | CWM1:PTV | 1 | 1866 | 98.6 | **<0.001** |
| PTVCWM2 | CWM2 | 1 | 121933 | 8002 | **<0.001** |
|  | PTV | 1 | 2835 | 186 | **<0.001** |
|  | CWM2:PTV | 1 | 2627 | 172 | **<0.001** |
| PTVCWM3 | CWM3 | 1 | 5232 | 94.6 | **<0.001** |
|  | PTV | 1 | 5652 | 102.2 | **<0.001** |
|  | CWM3:PTV | 1 | 7241 | 131 | **<0.001** |
| PTVFD1 | FD1 | 1 | 3626 | 62.4 | **<0.001** |
|  | PTV | 1 | 6069 | 104.4 | **<0.001** |
|  | FD1:PTV | 1 | 675 | 11.6 | **<0.001** |
| PTVFD2 | FD2 | 1 | 74932 | 2326.8 | **<0.001** |
|  | PTV | 1 | 3088 | 95.9 | **<0.001** |
|  | FD2:PTV | 1 | 3090 | 96 | **<0.001** |
| PTVFD3 | FD3 | 1 | 2 | 0.03 | 0.85 |
|  | PTV | 1 | 7684 | 131.26 | **<0.001** |
|  | FD3:PTV | 1 | 1569 | 26.8 | **<0.001** |

**Table S7** Spatial simultaneous autoregressive (SAR) error models ANOVA table for the interactive effect of functional diversity and mean annual temperature (MAT) on NPP. P ≤ 0.05 are in boldface.

|  | **Model** | **DF** | **AIC** | **logLik** | **L.Ratio** | ***P* value** |
| --- | --- | --- | --- | --- | --- | --- |
| MATCWM1 | matcw1 | 6 | 10592 | -5290 |  |  |
|  | withoutcw1XMAT | 5 | 10607 | -5299 | 17.3 | **<0.001** |
| MATCWM2 | matcw2 | 6 | 10516 | -5252 |  |  |
|  | matwithoutcw2XMAT | 5 | 10571 | -5280 | 56.7 | **<0.001** |
| MATCWM3 | matcw3 | 6 | 10596 | -5292 |  |  |
|  | matwithoutcw3XMAT | 5 | 10594 | -5292 | 0.00395 | 0.95 |
| MATFD1 | matfd1 | 6 | 10611 | -5300 |  |  |
|  | matwithoutfd1XMAT | 5 | 10614 | -5302 | 4.59 | **0.0322** |
| MATFD2 | matfd2 | 6 | 10580 | -5284 |  |  |
|  | matwithoutfd2XMAT | 5 | 10602 | -5296 | 23.3 | **<0.001** |
| MATFD3 | matfd3 | 6 | 10611 | -5300 |  |  |
|  | matwithoutfd3XMAT | 5 | 10610 | -5300 | 1.36 | 0.244 |

**Table S8** Spatial simultaneous autoregressive (SAR) error models ANOVA table for the interactive effect of functional diversity and mean annual precipitation (MAP) on NPP. P ≤ 0.05 are in boldface.

|  | **Model** | **DF** | **AIC** | **logLik** | **L.Ratio** | ***P* value** |
| --- | --- | --- | --- | --- | --- | --- |
| MAPCWM1 | mapcw1 | 6 | 10509 | -5249 |  |  |
|  | mapwithoutcw1Xmap | 5 | 10509 | -5249 | 1.59 | 0.207 |
| MAPCWM2 | mapcw2 | 6 | 10473 | -5230 |  |  |
|  | mapwithoutcw2Xmap | 5 | 10482 | -5236 | 10.9 | **<0.001** |
| MAPCWM3 | mapcw3 | 6 | 10514 | -5251 |  |  |
|  | mapwithoutcw3Xmap | 5 | 10513 | -5251 | 1.01 | 0.316 |
| MAPFD1 | mapfd1 | 6 | 10519 | -5253 |  |  |
|  | mapwithoutfd1Xmap | 5 | 10521 | -5255 | 3.55 | 0.0594 |
| MAPFD2 | mapfd2 | 6 | 10505 | -5247 |  |  |
|  | mapwithoutfd2Xmap | 5 | 10511 | -5251 | 8.29 | **0.004** |
| MAPFD3 | mapfd3 | 6 | 10519 | -5253 |  |  |
|  | mapwithoutfd3Xmap | 5 | 10519 | -5254 | 2.01 | 0.156 |

**Table S9** Spatial simultaneous autoregressive (SAR) error models ANOVA table for the interactive effect of functional diversity and paleo-temperature velocity (PTV) on NPP. P ≤ 0.05 are in boldface.

|  | **Model** | **DF** | **AIC** | **logLik** | **L.Ratio** | ***P* value** |
| --- | --- | --- | --- | --- | --- | --- |
| PTVCWM1 | ptvcw1 | 6 | 10632 | -5310 |  |  |
|  | ptvwithoutcw1Xptv | 5 | 10630 | -5310 | 0.0311 | 0.86 |
| PTVCWM2 | ptvcw2 | 6 | 10601 | -5294 |  |  |
|  | ptvwithoutcw2Xptv | 5 | 10600 | -5295 | 1.44 | 0.23 |
| PTVCWM3 | ptvcw3 | 6 | 10631 | -5309 |  |  |
|  | ptvwithoutcw3Xptv | 5 | 10629 | -5309 | 0.011 | 0.916 |
| PTVFD1 | ptvfd1 | 6 | 10642 | -5315 |  |  |
|  | ptvwithoutfd1Xptv | 5 | 10642 | -5316 | 1.32 | 0.25 |
| PTVFD2 | ptvfd2 | 6 | 10632 | -5310 |  |  |
|  | ptvwithoutfd2Xptv | 5 | 10631 | -5310 | 1.18 | 0.278 |
| PTVFD3 | ptvfd3 | 6 | 10638 | -5313 |  |  |
|  | ptvwithoutfd3Xptv | 5 | 10638 | -5314 | 2.55 | 0.11 |

**Table S10** SEM model output for plant size functional diversity effects on NPP. P ≤ 0.05 are in boldface.

| **Response ~ predictor** | **Estimate** | **SE** | ***P*** | **Stand.Est** |
| --- | --- | --- | --- | --- |
| **FD1~** |  |  |  |  |
| MAT | 0.0038 | 0.000 | **<0.001** | 0.26 |
| MAP | -0.0001 | 0.000 | **<0.001** | -0.46 |
| PTV | 0.0012 | 0.000 | **<0.001** | 0.08 |
| **CWM1~** |  |  |  |  |
| MAT | 0.0197 | 0.000 | **<0.001** | 0.46 |
| MAP | 0.0002 | 0.000 | **<0.001** | 0.38 |
| PTV | -0.0014 | 0.000 | **<0.001** | -0.03 |
| **NPP~** |  |  |  |  |
| FD1 | 3.4168 | 0.727 | **<0.001** | 0.06 |
| CWM1 | 6.9421 | 0.382 | **<0.001** | 0.33 |
| MAT | -0.0592 | 0.018 | **<0.001** | -0.07 |
| MAP | 0.0077 | 0.000 | **<0.001** | 0.59 |
| PTV | -0.1164 | 0.012 | **<0.001** | -0.12 |
|  |  |  |  |  |
| **Covariance** |  |  |  |  |
| **Variables** | **Estimate** | **P** | **Stand.Est** |  |
| **FD1~~** |  |  |  |  |
| CWM1 | -0.06 | **0.001** | -0.06 |  |
|  |  |  |  |  |
| ***R^2^*** |  |  |  |  |
| **Variable** | **Estimate** |  |  |  |
| FD1 | 0.12 |  |  |  |
| CWM1 | 0.63 |  |  |  |
| NPP | 0.71 |  |  |  |

**Table S11** SEM model output for leaf morphology functional diversity effects on NPP. P ≤ 0.05 are in boldface.

| **Response ~ predictor** | **Estimate** | **SE** | ***P*** | **Stand.Est** |
| --- | --- | --- | --- | --- |
| **FD2~** |  |  |  |  |
| MAT | 0.0025 | 0.000 | **<0.001** | 0.12 |
| MAP | -0.0002 | 0.000 | **<0.001** | -0.77 |
| PTV | -0.0016 | 0.000 | **<0.001** | -0.07 |
| **CWM2~** |  |  |  |  |
| MAT | 0.0033 | 0.001 | **0.009** | 0.05 |
| MAP | 0.0006 | 0.000 | **<0.001** | 0.75 |
| PTV | 0.0047 | 0.000 | **<0.001** | 0.07 |
| **NPP~** |  |  |  |  |
| FD2 | 1.6865 | 0.765 | **0.028** | 0.04 |
| CWM2 | 7.1818 | 0.326 | **<0.001** | 0.48 |
| MAT | 0.0628 | 0.015 | **<0.001** | 0.07 |
| MAP | 0.0048 | 0.000 | **<0.001** | 0.37 |
| PTV | -0.1529 | 0.011 | **<0.001** | -0.16 |
|  |  |  |  |  |
| **Covariance** |  |  |  |  |
| **Variables** | **Estimate** | **P** | **Stand.Est** |  |
| **FD2~~** | -0.67 | **<0.001** | -0.67 |  |
| CWM2 |  |  |  |  |
|  |  |  |  |  |
| ***R^2^*** |  |  |  |  |
| **Variable** | **Estimate** |  |  |  |
| FD2 | 0.44 |  |  |  |
| CWM2 | 0.59 |  |  |  |
| NPP | 0.75 |  |  |  |

**Table S12** SEM model output for flower duration functional diversity effects on NPP. P ≤ 0.05 are in boldface.

| **Response ~ predictor** | **Estimate** | **SE** | ***P*** | **Stand.Est** |
| --- | --- | --- | --- | --- |
| **FD3~** |  |  |  |  |
| MAT | 0.0014 | 0.000 | **<0.001** | 0.11 |
| MAP | 0.0001 | 0.000 | **<0.001** | 0.33 |
| PTV | 0.0008 | 0.000 | **0.006** | 0.06 |
| **CWM3~** |  |  |  |  |
| MAT | 0.0262 | 0.000 | **<0.001** | 0.79 |
| MAP | -0.0001 | 0.000 | **<0.001** | -0.11 |
| PTV | -0.0052 | 0.000 | **<0.001** | -0.14 |
| **NPP~** |  |  |  |  |
| FD3 | 6.3339 | 0.976 | **<0.001** | 0.09 |
| CWM3 | -5.6996 | 0.502 | **<0.001** | -0.21 |
| MAT | 0.2309 | 0.021 | **<0.001** | 0.26 |
| MAP | 0.0083 | 0.000 | **<0.001** | 0.64 |
| PTV | -0.1569 | 0.013 | **<0.001** | -0.16 |
|  |  |  |  |  |
| **Covariance** |  |  |  |  |
| **Variables** | **Estimate** | **P** | **Stand.Est** |  |
| **FD3~~** |  |  |  |  |
| CWM3 | 0.40 | **<0.001** | 0.40 |  |
|  |  |  |  |  |
| ***R^2^*** |  |  |  |  |
| **Variable** | **Estimate** |  |  |  |
| FD3 | 0.17 |  |  |  |
| CWM3 | 0.54 |  |  |  |
| NPP | 0.68 |  |  |  |

**Table S13** SEM model output for three axes of woody vegetation functional diversity effects on NPP. P ≤ 0.05 are in boldface.

| **Response ~ predictor** | **Estimate** | **SE** | ***P*** | **Stand.Est** |
| --- | --- | --- | --- | --- |
| **FD1~** |  |  |  |  |
| MAT | 0.0038 | 0.001 | **<0.001** | 0.26 |
| MAP | -0.0001 | 0.000 | **<0.001** | -0.46 |
| PTV | 0.0012 | 0.000 | **<0.001** | 0.08 |
| **FD2~** |  |  |  |  |
| MAT | 0.0025 | 0.001 | **<0.001** | 0.12 |
| MAP | -0.0002 | 0.000 | **<0.001** | -0.77 |
| PTV | -0.0016 | 0.000 | **<0.001** | -0.07 |
| **FD3~** |  |  |  |  |
| MAT | 0.0014 | 0.000 | **<0.001** | 0.11 |
| MAP | 0.0001 | 0.000 | **<0.001** | 0.33 |
| PTV | 0.0008 | 0.000 | **0.006** | 0.06 |
| **CWM1~** |  |  |  |  |
| MAT | 0.0197 | 0.001 | **<0.001** | 0.46 |
| MAP | 0.0002 | 0.000 | **<0.001** | 0.38 |
| PTV | -0.0014 | 0.001 | **0.027** | -0.03 |
| **CWM2~** |  |  |  |  |
| MAT | 0.0033 | 0.001 | **0.009** | 0.05 |
| MAP | 0.0006 | 0.000 | **<0.001** | 0.75 |
| PTV | 0.0047 | 0.001 | **<0.001** | 0.07 |
| **CWM3~** |  |  |  |  |
| MAT | 0.0262 | 0.001 | **<0.001** | 0.79 |
| MAP | -0.0001 | 0.000 | **<0.001** | -0.11 |
| PTV | -0.0052 | 0.001 | **<0.001** | -0.14 |
| **NPP~** |  |  |  |  |
| FD1 | 2.651 | 0.675 | **<0.001** | 0.04 |
| FD3 | 8.1176 | 0.886 | **<0.001** | 0.12 |
| CWM1 | 1.6205 | 0.414 | **<0.001** | 0.08 |
| CWM2 | 6.9878 | 0.331 | **<0.001** | 0.47 |
| CWM3 | 0.8063 | 0.376 | **0.032** | 0.03 |
| MAP | 0.0038 | 0.000 | **<0.001** | 0.29 |
| PTV | -0.1589 | 0.011 | **<0.001** | -0.16 |
|  |  |  |  |  |
| **Covariance** |  |  |  |  |
| **Variables** | **Estimate** | **P** | **Stand.Est** |  |
| **FD1~~** |  |  |  |  |
| FD2 | 0.2139 | **<0.001** | 0.21 |  |
| FD3 | 0.2634 | **<0.001** | 0.26 |  |
| CWM1 | -0.0624 | **0.001** | -0.06 |  |
| CWM2 | -0.093 | **<0.001** | -0.09 |  |
| CWM3 | 0.0585 | **0.002** | 0.06 |  |
| **FD2~~** |  |  |  |  |
| FD3 | 0.1622 | **<0.001** | 0.16 |  |
| CWM1 | -0.3127 | **<0.001** | -0.31 |  |
| CWM2 | -0.6668 | **<0.001** | -0.67 |  |
| CWM3 | 0.3274 | **<0.001** | 0.33 |  |
| **FD3~~** |  |  |  |  |
| CWM1 | -0.3312 | **<0.001** | -0.33 |  |
| CWM2 | -0.2799 | **<0.001** | -0.28 |  |
| CWM3 | 0.3961 | **<0.001** | 0.40 |  |
| **CWM1~~** |  |  |  |  |
| CWM2 | 0.6625 | **<0.001** | 0.66 |  |
| CWM3 | -0.4923 | **<0.001** | -0.49 |  |
| **CWM2~~** |  |  |  |  |
| CWM3 | -0.5227 | **<0.001** | -0.52 |  |
|  |  |  |  |  |
| ***R^2^*** |  |  |  |  |
| **Variable** | **Estimate** |  |  |  |
| FD1 | 0.12 |  |  |  |
| FD2 | 0.44 |  |  |  |
| FD3 | 0.17 |  |  |  |
| CWM1 | 0.63 |  |  |  |
| CWM2 | 0.59 |  |  |  |
| CWM3 | 0.54 |  |  |  |
| NPP | 0.76 |  |  |  |

**Table S14** SEM model output for three axes of woody vegetation functional diversity effects on biomass. P ≤ 0.05 are in boldface.

| **Response ~ predictor** | **Estimate** | **SE** | ***P*** | **Stand.Est** |
| --- | --- | --- | --- | --- |
| **FD1~** |  |  |  |  |
| MAT | 0.0038 | 0.001 | **<0.001** | 0.26 |
| MAP | -0.0001 | 0.000 | **<0.001** | -0.46 |
| PTV | 0.0012 | 0.000 | **<0.001** | 0.08 |
| **FD2~** |  |  |  |  |
| MAT | 0.0025 | 0.001 | **<0.001** | 0.12 |
| MAP | -0.0002 | 0.000 | **<0.001** | -0.77 |
| PTV | -0.0016 | 0.000 | **<0.001** | -0.07 |
| **FD3~** |  |  |  |  |
| MAT | 0.0014 | 0.000 | **<0.001** | 0.11 |
| MAP | 0.0001 | 0.000 | **<0.001** | 0.33 |
| PTV | 0.0008 | 0.000 | **0.006** | 0.06 |
| **CWM1~** |  |  |  |  |
| MAT | 0.0197 | 0.001 | **<0.001** | 0.46 |
| MAP | 0.0002 | 0.000 | **<0.001** | 0.38 |
| PTV | -0.0014 | 0.001 | **0.027** | -0.03 |
| **CWM2~** |  |  |  |  |
| MAT | 0.0033 | 0.001 | **0.009** | 0.05 |
| MAP | 0.0006 | 0.000 | **<0.001** | 0.75 |
| PTV | 0.0047 | 0.001 | **<0.001** | 0.07 |
| **CWM3~** |  |  |  |  |
| MAT | 0.0262 | 0.001 | **<0.001** | 0.79 |
| MAP | -0.0001 | 0.000 | **<0.001** | -0.11 |
| PTV | -0.0052 | 0.001 | **<0.001** | -0.14 |
| **Biomass~** |  |  |  |  |
| FD2 | 159800.00 | 22056.479 | **<0.001** | 0.19 |
| FD3 | 174300.00 | 25201.413 | **<0.001** | 0.12 |
| CWM2 | 43170.00 | 10448.042 | **<0.001** | 0.14 |
| CWM3 | -162100.00 | 14582.238 | **<0.001** | -0.29 |
| MAT | -2175.00 | 581.466 | **<0.001** | -0.12 |
| MAP | 196.10 | 8.543 | **<0.001** | 0.72 |
| PTV | -6393.00 | 324.969 | **<0.001** | -0.31 |
|  |  |  |  |  |
| **Covariance** |  |  |  |  |
| **Variables** | **Estimate** | **P** | **Stand.Est** |  |
| **FD1~~** |  |  |  |  |
| FD2 | 0.2139 | **<0.001** | 0.21 |  |
| FD3 | 0.2634 | **<0.001** | 0.26 |  |
| CWM1 | -0.0624 | **0.001** | -0.06 |  |
| CWM2 | -0.0930 | **<0.001** | -0.09 |  |
| CWM3 | 0.0585 | **0.002** | 0.06 |  |
| **FD2~~** |  |  |  |  |
| FD3 | 0.1622 | **<0.001** | 0.16 |  |
| CWM1 | -0.3127 | **<0.001** | -0.31 |  |
| CWM2 | -0.6668 | **<0.001** | -0.67 |  |
| CWM3 | 0.3274 | **<0.001** | 0.33 |  |
| **FD3~~** |  |  |  |  |
| CWM1 | -0.3312 | **<0.001** | -0.33 |  |
| CWM2 | -0.2799 | **<0.001** | -0.28 |  |
| CWM3 | 0.3961 | **<0.001** | 0.40 |  |
| **CWM1~~** |  |  |  |  |
| CWM2 | 0.6625 | **<0.001** | 0.66 |  |
| CWM3 | -0.4923 | **<0.001** | -0.49 |  |
| **CWM2~~** |  |  |  |  |
| CWM3 | -0.5227 | **<0.001** | -0.52 |  |
|  |  |  |  |  |
| ***R^2^*** |  |  |  |  |
| **Variable** | **Estimate** |  |  |  |
| FD1 | 0.12 |  |  |  |
| FD2 | 0.44 |  |  |  |
| FD3 | 0.17 |  |  |  |
| CWM1 | 0.63 |  |  |  |
| CWM2 | 0.59 |  |  |  |
| CWM3 | 0.54 |  |  |  |
| Biomass | 0.53 |  |  |  |
